# The role of R-loop aberrations in lower-grade gliomas: prognostic, immune, and metabolic implications from multi-omics and machine learning analysis

**DOI:** 10.3389/fimmu.2026.1758954

**Published:** 2026-04-17

**Authors:** Ronghua Huang, Bing-Biao Lin, Hongxin Huang, Chenrui Li, Shaohui Zhuang, Naili Wei, Yuanfeng Yu, Wenfei Zhou, Yue Qiu, Zhijie Lu, Yixuan Hao, Zixian Lin, Chuangzhen Chen, Jian Chen

**Affiliations:** 1 Department of Neurosurgery, The First Affiliated Hospital of Shantou University Medical College, Shantou, Guangdong, China; 2 Department of Anesthesiology, The First Affiliated Hospital of Shantou University Medical College, Shantou, Guangdong, China; 3 Department of Radiation Oncology, Cancer Hospital of Shantou University Medical College, Shantou, Guangdong, China; 4 Department of Obstetrics, Rongcheng Women Infant Health Care Hospital, Jieyang, Guangdong, China

**Keywords:** lower-grade gliomas, metabolism, prognosis, R-loop, tumor microenvironment

## Abstract

**Background:**

The prognostic and therapeutic implications of R-loop regulators in lower-grade glioma (LGG) remain largely unexplored.

**Methods:**

We constructed a R-loop prognostic index (RLPI) by benchmarking 9 machine-learning algorithms through nested cross-validation. Associations between RLPI and clinical outcomes, immune microenvironment, and metabolic features were assessed across –11 bulk transcriptomic, 2 single-cell RNA-sequencing, and 1 spatial transcriptomic LGG cohorts. Functional validation was performed for key RLPI genes.

**Results:**

Among the 9 algorithms tested, Elastic Net demonstrated the best predictive performance and was selected to construct the RLPI, which comprised 35 R-loop regulators. Elevated RLPI robustly predicted poorer prognosis in 1556 patients across 6 independent LGG cohorts. High-RLPI glioma cells were enriched in angiogenesis and hypoxia pathways and exhibited a mesenchymal-like state with heightened metabolic activity. Notably, RLPI correlated with enhanced antigen presentation and immunosuppressive signaling, alongside increased infiltration of immune cells with pro- or anti-tumor functions. Moreover, high RLPI correlated with resistance to radiotherapy and temozolomide but improved response to immune checkpoint blockade therapy. Functional assays revealed that knockdown of INCENP suppressed glioma cell proliferation, migration, and invasion while promoting apoptosis, whereas NCAPG silencing impaired migration and invasion.

**Conclusion:**

This study establishes the RLPI as a promising biomarker for personalized risk stratification and treatment guidance in LGG.

## Highlights

- A novel R-loop prognostic index (RLPI) was developed to reliably predict clinical outcomes in patients with lower-grade glioma (LGG).
- High RLPI scores are associated with a mesenchymal-like tumor phenotype and enhanced metabolic activity in LGG.
- Elevated RLPI is linked to increased immune cell infiltration and intensified immune interactions within the tumor microenvironment.

## 1 Introduction

Lower-grade gliomas (LGGs) are heterogeneous neuroepithelial neoplasms arising from glial cells of the central nervous system (CNS) and encompass World Health Organization (WHO) grades I-III, thereby distinguishing them from glioblastoma (GBM, grade IV) (1, 2). LGGs account for approximately 30% of pediatric and 6.4% of adult primary CNS tumors, with overall survival averaging approximately 7 years (3, 4). Standard treatment for LGG typically involves surgical resection, occasionally followed by chemotherapy or radiotherapy (1). Despite these interventions, a significant proportion of LGGs progress to higher-grade tumors, leading to poor clinical outcomes and diminished quality of life (1, 2, 4). Consequently, substantial research efforts are currently directed toward investigating actionable oncogenic mechanisms and identifying effective biomarkers (5–8). While clinicopathological factors such as tumor size and age at diagnosis are currently used to estimate the risk of LGG progression and therapeutic failure (1), these factors are limited by insufficient accuracy and fail to reflect the molecular heterogeneity within and across tumors (4). Therefore, there is an urgent need to identify robust molecular biomarkers that can accurately pinpoint high-risk LGG patients and guide timely and personalized therapeutic strategies.

R-loops are defined as three-stranded nucleic acid structures comprising a DNA: RNA hybrid and a displaced single-stranded DNA loop, typically formed during transcription or through the pairing of non-coding RNAs with complementary DNA sequences (9, 10). R-loops are widespread across the genome and are involved in essential biological processes, including telomere stability, DNA methylation, mitochondrial DNA replication, and transcription regulation (9, 10). However, aberrant formation or resolution of R-loops can impair these regulatory functions and induce DNA replication stress and genomic instability (10–12). Such dysregulation has been increasingly implicated in various human diseases (13–15), particularly cancer, where it may contribute to oncogene activation, tumor suppressor gene inactivation, and immune evasion (10, 13, 16, 17). To date, an expanding repertoire of R-loop regulatory factors has been identified and functionally characterized, revealing their ability to modulate the stability, localization, and abundance of R-loops (18–20). Nonetheless, the roles of these R-loop regulators as biomarkers for disease progression and prognosis in LGG remain unexplored.

To address these gaps, this study leveraged R-loop regulators to develop and validate a robust R-loop related prognostic index (RLPI) in 1556 LGG patients from 6 independent cohorts that could effectively identify patients with worse prognosis. Furthermore, integrative analyses using single-cell RNA sequencing (scRNA-seq) and spatial transcriptomics (ST) revealed that a high RLPI score was associated with a mesenchymal-like transcriptional state in glioma cells, as well as with distinct alterations in metabolism, tumor microenvironment (TME) composition, and intercellular communication patterns.

## 2 Method

### 2.1 Collection and processing of bulk transcriptomic data

This study included only LGG samples with available overall survival data. Transcriptomic expression data generated by the Illumina HiSeq platform and corresponding clinical information for the TCGA-LGG (n = 506) and Chinese Glioma Genome Atlas-LGG (CGGA-LGG, n = 605) cohorts were obtained from the UCSC Xena platform (https://xena.ucsc.edu/) and the CGGA (http://www.cgga.org.cn/), respectively (2, 21). Additionally, 4 LGG microarray datasets, including Gravendeel (n = 115), Gorovets (n = 65), Kamoun (n = 126), along with the Rembrandt (n = 139), were collected from the Gene Expression Omnibus (GEO, https://www.ncbi.nlm.nih.gov/geo/) (22–25). TCGA-LGG was used for model development, while the remaining 5 LGG datasets were used for external validation. Also, transcriptomic and clinical data of 7 GBM cohorts and 4 pan-glioma cohorts were collected from the GlioVis data portal (https://gliovis.bioinfo.cnio.es/).

For RNA sequencing datasets, gene expression values were calculated as log_2_(normalized count + 0.5). For Affymetrix microarray data, background correction and normalization were performed using the Robust Multi-array Average (RMA) algorithm via the affy R package, followed by quantile normalization. When multiple probe sets corresponded to the same gene, the median expression value across probes was used.

Additionally, we retrieved transcriptomic and clinical data from 4 cohorts treated with anti-PD-L1 agents, including IMvigor210 (atezolizumab in urothelial carcinoma) (26), GSE173839 (durvalumab in breast cancer) (27), and the Nabet and Finn cohorts (accessed through https://cide.ccr.cancer.gov/) (28, 29). We also included the Nathanson cohort (30), which assessed response to anti-CTLA-4 therapy in melanoma.

### 2.2 R- loop related gene feature selection

We firstly retrieved 1185 R-loop regulatory genes from a prior study (31), and excluded those not detected in the LGG bulk RNA-seq datasets. We then used univariate Cox regression to identify genes significantly linked to LGG prognosis (P < 0.01) in the TCGA-LGG training cohort. To enhance selection robustness, we employed a bootstrap approach (1000 resamples of 80% patients) and retained only genes achieving the P < 0.01 threshold in over 800 iterations. Finally, we applied the Boruta algorithm (ntree=1000, maxRuns=1000) to filter for genes deemed most prognostically relevant based on feature importance compared to random features.

### 2.3 Construction of a R-loop related prognostic index

To develop a robust and precise R-loop associated model, we implemented a nested cross-validation (CV) approach to evaluate 9 machine learning algorithms: Elastic Net (Enet), Ridge Regression, Least Absolute Shrinkage and Selection Operator (Lasso), Partial Least Squares Regression for Cox (plsRcox), CoxBoost, Generalized Boosted Regression Modeling (GBoost), eXtreme Gradient Boosting Survival (XGBoost), Random Survival Forest (RSF), and Supervised Principal Components (SuperPC) (32). The TCGA-LGG dataset was partitioned into 10 approximately equal-sized folds, with each fold serving as the test set and the remaining nine folds forming the training set. Hyperparameter optimization for each algorithm was conducted using a nested 5-fold CV within the training set, with hyperparameter ranges detailed in **Supplementary Table S1**. To ensure model reliability, the ratio of surviving to deceased patients was maintained consistently across all folds. Each algorithm was trained on the training set using the hyperparameters that yielded optimal performance in the inner CV and was subsequently evaluated on the corresponding test set. Model performance was quantified using Harrell’s Concordance Index (C-index), the Integrated Brier Score (IBS), and the time-dependent area under the receiver operating characteristic (AUC) across the 10 test sets. The optimal model was determined as the one exhibiting the highest average C-index and AUC, combined with the lowest average IBS.

### 2.4 Collection and analysis of single-cell RNA sequencing data

Two scRNA-seq datasets, GSE222850 and GSE244433, were curated (33, 34), resulting in a total of 10 lower-grade glioma (LGG) samples after excluding one neuronal tumor sample. Single-cell analyses were performed using the Seurat R package on filtered feature count matrices. Low-quality cells expressing fewer than 500 genes or with >20% mitochondrial gene content were excluded. Genes expressed in fewer than three cells were also removed. Potential doublets were identified and removed using DoubletFinder.

Following quality control, raw counts were normalized using the LogNormalize function. Principal component analysis was conducted on the top 2,000 most variable genes identified by the FindVariableFeatures function. The top 50 principal components were used for batch correction via Harmony, followed by graph-based Louvain clustering and visualization with Uniform Manifold Approximation and Projection (UMAP). Major cell types were annotated based on canonical markers. Clusters expressing mixed lineage markers were considered contaminated and excluded from further analysis. To distinguish malignant from non-malignant cells, genome-wide copy number variation profiles were inferred from gene expression data using the Bayesian segmentation algorithm CopyKat. Cells exhibiting aneuploidy were classified as tumor cells and further validated by the expression of known tumor markers.

### 2.5 Identification of malignant metaprograms via consensus non-negative matrix factorization

Consensus non-negative matrix factorization (cNMF) was employed to identify gene expression programs (GEPs) recurrently expressed across samples (35). Each cNMF run was repeated 50 times, and the optimal number of components (k = 2 - 30) was determined by balancing the silhouette stability score and Frobenius reconstruction error. Cross-sample GEP scores were then clustered using hierarchical clustering with “1 - Pearson correlation coefficient” as the distance metric and average linkage. The resulting dendrogram was pruned at an appropriate height to define metaprograms (MPs), with the target number set to 20. MPs with functional similarity were then consolidated to yield a consensus MP. To characterize each consensus MP, the top 50 genes with the highest loading values were selected. MP scores were then computed for each malignant cell using the UCell method. These scores were subsequently used for clustering glioma cells and visualizing their distribution via UMAP.

### 2.6 Assignment of GBM molecular subtypes to LGG cells

Glioma molecular subtypes were assigned to LGG cells based on the meta-modules defined by Neftel et al. (36), which include MES1-like, MES2-like, NPC1-like, NPC2-like, AC-like, and OPC-like states. For analytical simplicity, MES1 and MES2 were consolidated into a single MES-like group, and NPC1 and NPC2 were merged into a single NPC-like group. Meta-module scores were calculated using the”sigScores” function from the scalop R package. Two-dimensional cellular state plots were generated, with each quadrant corresponding to a distinct molecular subtype.

### 2.7 Metabolic landscape modeling using compass

Compass analysis was performed to infer the metabolic landscape at single-cell resolution using standard parameters (37). The underlying metabolic network was derived from the Recon2 database, and core metabolic reactions were defined according to previously described workflows. Compass scores were calculated for each reaction across individual cells. To identify differentially active reactions between cell subpopulations, we applied the Wilcoxon rank-sum test to compare Compass scores between groups. Effect sizes were estimated using Cohen’s d statistic to quantify the magnitude of differences in reaction activity. Resulting P values were adjusted for multiple comparisons using the Benjamini-Hochberg method. Reactions with an adjusted P value < 0.05 were considered significantly differentially active.

### 2.8 Cell-cell communication analysis using CellChat

CellChat was employed to identify differential signaling pathways and interaction strengths associated with RLPI status. Toward this end, we first separated LGG samples into RLPI-high and RLPI-low groups based on the median RLPI score. For each group, CellChat objects were created using the createCellChat function, and communication probabilities were inferred using computeCommunProb. The two CellChat objects were then merged via the mergeCellChat function to enable comparative analysis. Differences in outgoing and incoming signaling patterns among cell types between the RLPI-high and RLPI-low groups were assessed using the compareInteractions, netAnalysis_signalingChanges_scatter, and netVisual_bubble functions.

### 2.9 Spatial transcriptomic analysis

Spatial transcriptomic (ST) data for LGG were obtained from the GSE270355 dataset and analyzed using the Seurat R package (38). To infer the spatial cellular composition of each spot, the Robust Cell Type Decomposition (RCTD) algorithm was applied with doublet_mode set to “full”, using the LGG scRNA-seq data as a reference. The spatial distribution of inferred cell types was visualized using the FeaturePlot function. Spatial ligand-receptor expression patterns were identified using the SpaGene algorithm (39).

### 2.10 Pathway enrichment and immune infiltration analyses

For scRNA-seq data, differentially expressed genes were identified using the FindMarkers function in the Seurat R package. Gene set enrichment analysis (GSEA) was then performed using Hallmark gene sets (h.all.v7.4.symbols) obtained from the Molecular Signatures Database (MSigDB; https://www.gsea-msigdb.org/gsea/msigdb/). For bulk transcriptomic data, single-sample gene set enrichment analysis (ssGSEA) was conducted using the GSVA R package to calculate activity scores for Hallmark pathways, four GBM state pathways, and radiotherapy/temozolomide-resistance gene sets (40). Additionally, ssGSEA was employed to estimate the relative abundance of 28 tumor-infiltrating immune cell populations based on established gene signatures. The MCP-counter algorithm was also applied to quantify immune and stromal cell populations.

### 2.11 Cell culture and transfection

The human glioma cell line U251 and Hs683 were obtained from Wuhan Pricella Biotechnology Co., Ltd. and cultured in DMEM (Gibco) supplemented with 10% fetal bovine serum (Cat. No. F0193, Sigma) and 1% penicillin-streptomycin (Cat. No. 15140122, Gibco) at 37 °C in a humidified atmosphere containing 5% CO_2_.

Small interfering RNA (siRNA) targeting INCENP and NCAPG was synthesized by RiboBio (Guangzhou, China) and transiently transfected into cells using jetPRIME (Cat. No. 101000046, Polyplus-transfection), following the manufacturer’s instructions. The sequences for siINCENP and siNCAPG were 5’-GGAUGGAUCUGAAUAGCGA-3’ and 5’-GGAGUUCAUUCAUUACCUU-3’, respectively. Transfection efficiency was evaluated by quantitative RT-PCR, western blotting, and immunofluorescence.

### 2.12 RNA extraction and real−time quantitative PCR

Total RNA was extracted using the RNA-Quick Purification Kit (Cat. No. 400-100, Goonie, Guangzhou, China) according to the manufacturer’s instructions. RNA concentration and purity were measured using a NanoDrop 2000 spectrophotometer (Thermo Fisher Scientific, USA). One microgram of total RNA was reverse transcribed into cDNA using the HiScript^®^ IV All-in-One Ultra RT SuperMix for qPCR (Cat. No. R433, Vazyme, China). Real-time Quantitative PCR (qPCR) was performed using TB Green^®^ Premix Ex Taq™ II (Cat. No. RR820A, Takara, Japan) on the Roche LightCycler 480 system. Primers used in this study were as follows: INCENP-forward 5’-AGGCTCCTGAATGTTGAGGTGC-3’ and INCENP-reverse 5’-GTGTGCTGTTGGCAATCTCCGT-3’; NCAPG-forward 5’- GAGGCTGCTGTCGATTAAGGA-3’ and NCAPG-reverse 5’- AACTGTCTTATCATCCATCGTGC-3’; GAPDH-forward 5’-GTCTCCTCTGACTTCAACAGCG-3’, GAPDH-reverse 5’-ACCACCCTGTTGCTGTAGCCAA-3’.

### 2.13 Western blotting

Transfected glioma cells were washed with ice-cold PBS and lysed in RIPA buffer (Cat. No. P0013B, Beyotime) containing protease and phosphatase inhibitors (Cat. No. P1045, Beyotime). Lysates were sonicated, centrifuged at 12,000 × g for 15 min at 4 °C, and protein concentrations were determined using a BCA kit (Cat. No. P0012, Beyotime). Equal amounts of protein were mixed with loading buffer (Cat. No. P0015, Beyotime), boiled at 98 °C for 10 min, separated on 7.5% SDS-PAGE gels (Cat. No. PG111, Epizyme) and transferred to 0.22 μm polyvinylidene fluoride membranes (Millipore). Membranes were blocked with Fast Blocking Fluid (Cat. No. G2052, Servicebio) for 15 min at room temperature, incubated overnight at 4 °C with primary antibodies, washed with 0.1% TBST, and incubated with secondary antibodies for 1 h. Signals were detected using a Tanon imaging system and quantified with ImageJ. The primary antibodies were: anti-INCENP (Cat. No. 30388, ProMab), anti-BAX (Cat.No. A0207, Abclonal), anti-Bcl2 (Cat.No. A19693, Abclonal), anti-Cyto C (Cat.No. A4912, Abclonal), anti-PARP (Cat.No.A0942, Abclonal), anti-Caspase-9 (Cat.No. A2636, Abclonal), anti-β-ACTIN (Cat. No. ZB15001-HRP-100, servicebio), anti-GAPDH (Cat.No. ZB15004-HRP-100, servicebio), anti-α-Tubulin (Cat.No. 66031, Proteintech), and anti-Vinculin (Cat.No. 26520-1-AP, Proteintech).

### 2.14 Immunofluorescence

Transfected U251 cells were washed with PBS, fixed with 4% paraformaldehyde, permeabilized with 0.1% Triton X-100, and blocked with 5% bovine serum albumin for 1 hour at room temperature. Cells were then incubated overnight at 4 °C with anti-INCENP antibody (ProMab, Cat. No. 30388), followed by incubation with Alexa Fluor^®^ 488-conjugated goat anti-mouse IgG H&L (Abcam, Cat. No. ab150113) for 1 hour at room temperature. Nuclei were counterstained with DAPI. Fluorescence images were captured using a Leica TCS SP8 X confocal microscope.

### 2.15 Cell viability and colony formation assays

Transfected glioma cells were seeded into 96-well plates at a density of 1-3 × 10^3^ cells/well. Cell viability was assessed using the Cell Counting Kit-8 (CCK-8; Cat. No. K1018, Apexbio, USA). At the indicated time points (Days 1, 2, 3, and 4), 10 μL of CCK-8 solution was added to each well, followed by a 2-hour incubation. Absorbance at 450 nm was then measured using a microplate reader (BioTek). For the colony formation assay, transfected glioma cells were seeded into 6-well plates at a density of 1000 cells/well for the siNC group, 1000 cells/well for the siNCAPG group, and 3000 cells/well for the siINCENP group. Cells were cultured for 10 days, then fixed with 4% paraformaldehyde for 20 minutes and stained with 0.25% crystal violet for 30 mins. Colonies were imaged and then quantified using ImageJ software.

### 2.16 Annexin V-FITC/PI apoptosis detection assay

Glioma cells transfected with siNC or siINCENP were harvested 48 hours post-transfection, resuspended in 500 μL of binding buffer, and stained with Annexin V-FITC and propidium iodide (PI) using the Annexin V-FITC/PI Apoptosis Detection Kit (Cat. No. K2003, Apexbio, USA), according to the manufacturer’s instructions. Cells were then analyzed by a BD Accuri™ C6 Plus flow cytometer.

### 2.17 Transwell assay

Transwell migration and invasion assays were conducted using 8 μm pore size transwell chambers, with or without Matrigel coating (Labselect, Cat. No. 14342 and 14347, respectively). At 24 hours post-transfection, 200 μL of serum-free DMEM containing 3 × 10^4^ cells (for migration) or 5 × 10^4^ cells (for invasion) was added to the upper chamber. The lower chamber was filled with 800 μL of complete culture medium as a chemoattractant. After 36 hours of incubation, cells on the lower surface of the membrane were fixed with 4% paraformaldehyde, stained with 0.1% crystal violet, and imaged under a microscope at 10 × magnification. For each well, three to five randomly selected fields were photographed, and the number of migrated or invaded cells per field was quantified and statistically compared.

### 2.18 Statistical analysis

All statistical analyses and data visualizations were performed using GraphPad Prism (v7.0) and R software (v4.4.3). Kaplan-Meier survival curves were compared using the log-rank test, and hazard ratios (HRs) with 95% confidence intervals (CIs) were estimated via Cox proportional hazards regression (survival R package). AUC curves were generated using the survivalROC R package, while the C-index and IBS were calculated using the Hmisc and survcomp R packages, respectively. Pearson’s correlation was applied to assess associations between continuous variables. For comparisons between two groups, Student’s t-test was used unless otherwise specified. In box plots, the boxes represent the interquartile range (25th to 75th percentile), with the median indicated by the center line. All statistical tests were two-sided, and P < 0.05 was considered statistically significant.

## 3 Results

### 3.1 Study designs

**Figure 1:**
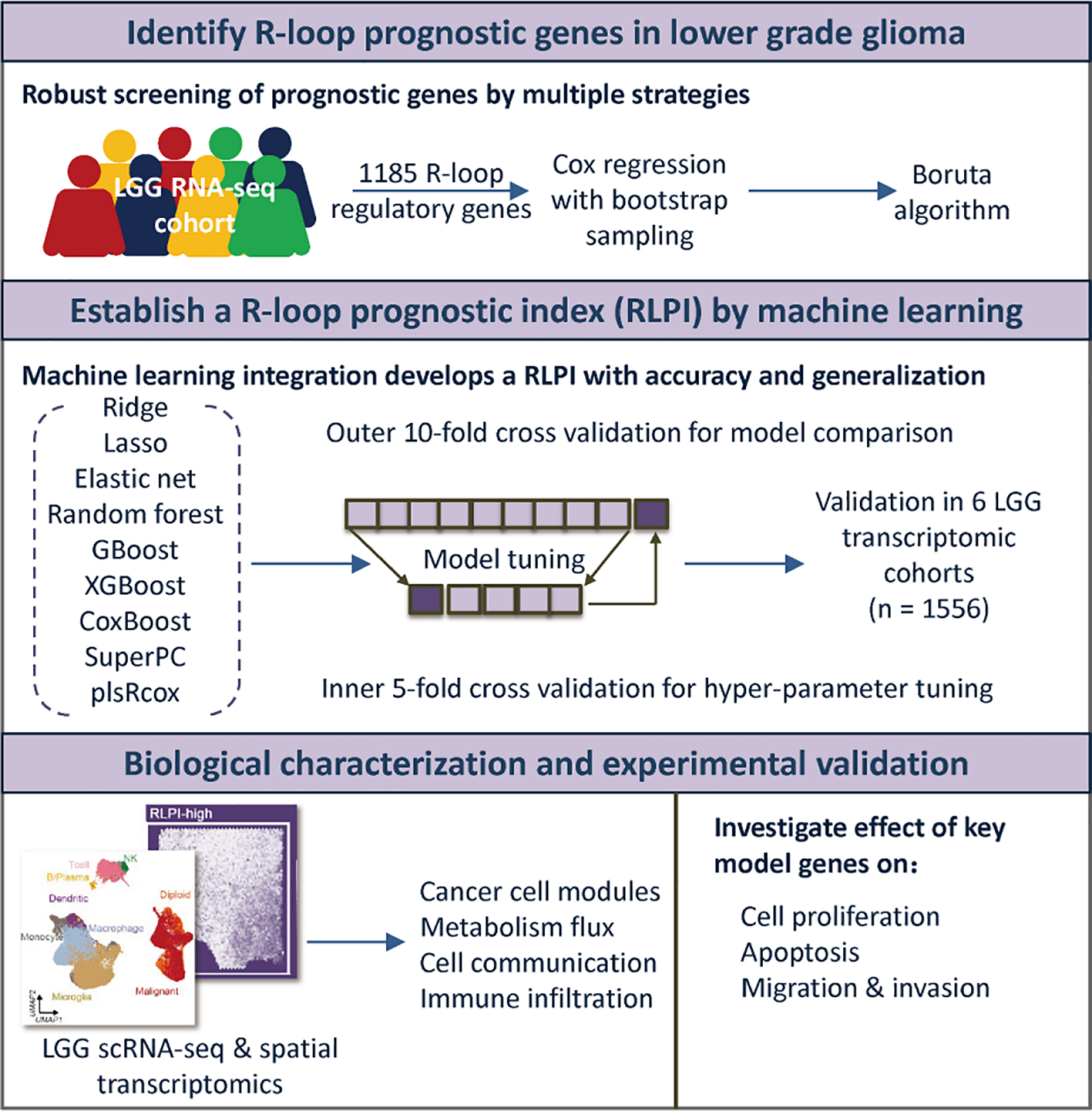
A schematic overview illustrating the workflow of this study. To comprehensively investigate the role of R-loop regulators in LGG progression, prognostic R-loop regulators were first identified. Subsequently, nine machine learning algorithms were benchmarked using a nested cross-validation framework to construct the optimal predictive model, referred to as the RLPI. The prognostic performance of the RLPI was validated across six independent LGG transcriptomic cohorts. Finally, integrative multi-omics analyses and functional experiments were conducted to elucidate the biological mechanisms associated with RLPI. LGG, lower-grade glioma; RLPI, R-loop related prognostic index; GBoost, generalized boosted regression modeling; XGBoost, eXtreme Gradient Boosting survival; SuperPC, supervised principal components; plsRcox, partial least squares regression for Cox; scRNA-seq, single-cell RNA sequencing.

A total of 1556 LGG cases with available survival data were included for model development and validation. The flowchart of this study is shown in **Figure 1**.

### 3.2 Selection of prognostic R-loop related features in TCGA-LGG

**Figure 2:**
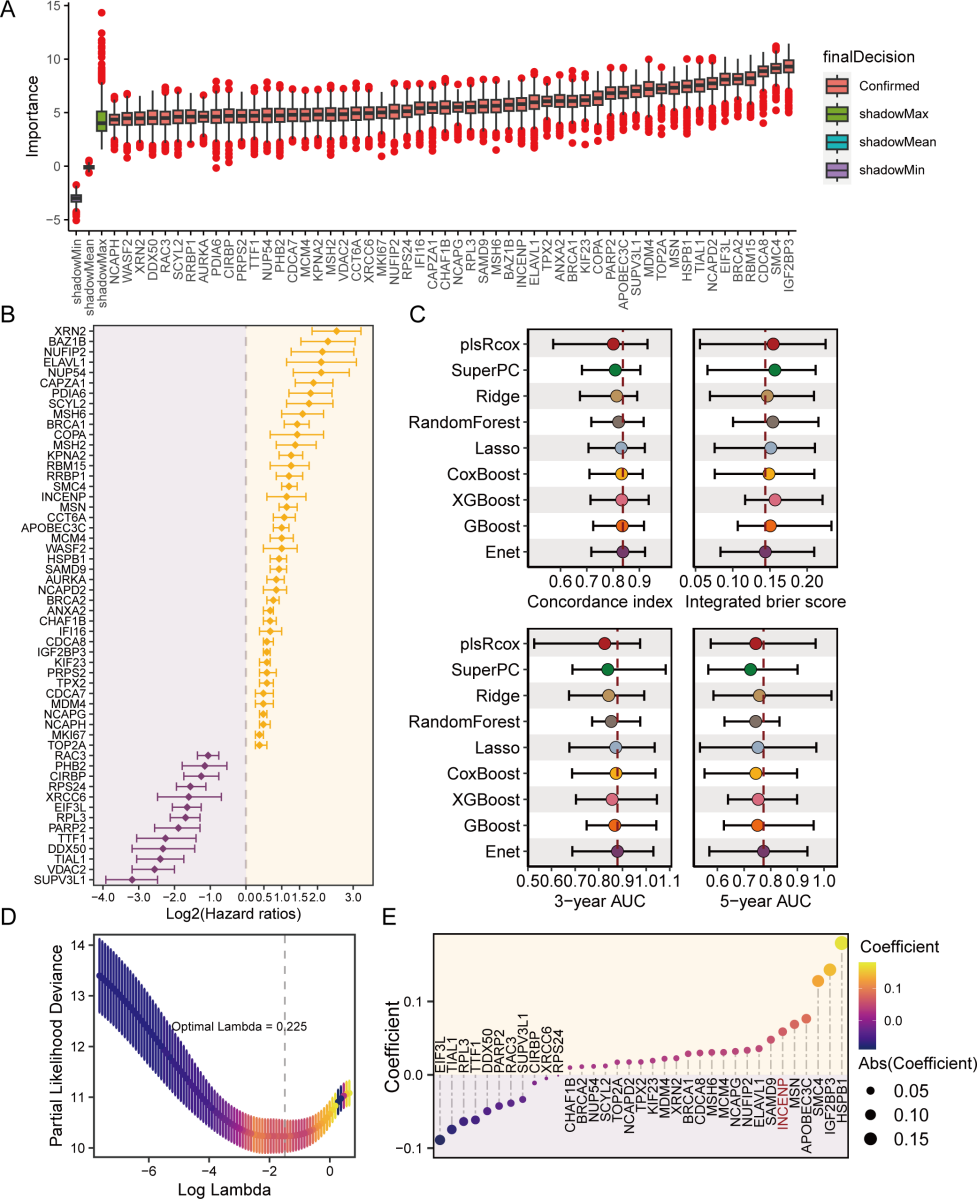
Development of a robust R-loop prognostic index for lower-grade glioma. **(A)** Boxplot showing the feature importance of prognostic R-loop regulatory genes identified by the Bortua algorithm. **(B)** Forest plot displaying the results of univariate Cox regression analysis for the 54 prognostic R-loop regulatory genes identified in **(A)**. **(C)** Harrell’s concordance index (C-index), integrated Brier score (IBS), and area under the receiver operating characteristic curve (AUC) values for 9 hyperparameter-tuned models were evaluated in an outer 10-fold cross-validation. Dots indicate each model’s mean of 10 C-indices, IBS, or AUC values. The model with the highest average C-index and AUC, and the lowest average IBS, was selected and defined as the R-loop prognostic index (RLPI). **(D)** Selection of the optimal regularization parameter (λ) for the elastic network model based on minimum partial likelihood deviance in the TCGA-LGG dataset. **(E)** Coefficients of the 35 RLPI genes inferred by the elastic network algorithm. Enet, elastic network; Lasso, Least Absolute Shrinkage and Selection Operator; plsRcox, partial least squares regression for Cox; GBoost, generalized boosted regression modeling; XGBoost, eXtreme Gradient Boosting survival; SuperPC, supervised principal components.

A total of 1185 R-loop regulatory genes were curated from a previous study, and 1050 were recovered in TCGA-LGG. Univariate Cox regression analysis identified 498 genes that were significantly related to overall survival in the TCGA-LGG datasets (**Supplementary Table S2**). The bootstrap approach further selected 389 of 498 prognostic genes that were robust to sample resampling (**Supplementary Figure S1A**, **Supplementary Table S3**). Furthermore, we adopted the Boruta algorithm and narrowed down the selected genes to 54 genes that were confirmed to reliably predict prognosis (**Supplementary Figure S1B**). As shown in **Figure 2A**, 54 genes were ranked according to the importance inferred by the Boruta algorithm. Among them, 41 genes are predictive of worse prognosis, while the remaining 13 are predictive of good prognosis (**Figure 2B**).

### 3.3 Development of a R-loop related prognostic index for LGG

Using features selected by the Boruta algorithm, we benchmarked 9 survival-related machine learning algorithms, including Enet, lasso, Ridge, XGBoost, plsRcox, SuperPC, CoxBoost, Random forest, and GBoost, to screen for a hyperparameter-tuned model with the best accuracy and lower risk of overfitting. To achieve this, a nested CV with outer 10 folds for validation and inner 5 folds for hyperparameter tuning was performed in the TCGA-LGG. As shown in **Figure 2C**; **Supplementary Table S4**, the Enet survival model achieved the best performance with the highest mean C-index (0.837), lowest mean IBS (0.144), and highest mean AUC values (3-year: 0.879; 5-year: 0.773). Therefore, the Enet model with tuned hyperparameters was then fitted to the entire TCGA-LGG dataset and referred to as RLPI (**Figure 2D**). The inferred feature contribution to RLPI was demonstrated in **Figure 2E**; **Supplementary Table S5**, with the top 3 features including HSPB1, IGF2BP3, and SMC4.

### 3.4 Evaluation of RLPI for risk stratification in LGG

**Figure 3:**
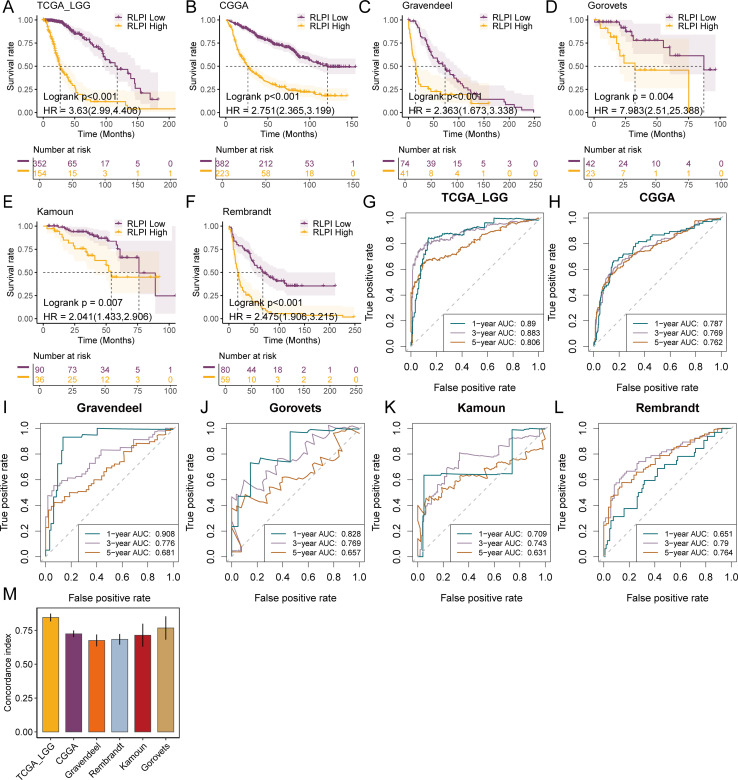
Validation of the R-loop prognostic index across 6 lower-grade glioma cohorts. **(A–F)** Kaplan-Meier survival curves comparing overall survival between high- and low-risk groups stratified by a universal RLPI threshold (0.137) in the TCGA-LGG **(A)**, CGGA-LGG **(B)**, Gravendeel **(C)**, Gorovets **(D)**, Kamoun **(E)**, and Rembrandt **(F)**. P values were derived from log-rank tests and were all < 0.01. Hazard ratios (HRs) and 95% confidence intervals (CIs) were calculated by univariate Cox regression analysis. **(G–L)** AUC curve analysis of RLPI for predicting prognosis at 1, 3, and 5 years in the cohorts of TCGA-LGG **(G)**, CGGA-LGG **(H)**, Gravendeel **(I)**, Gorovets **(J)**, Kamoun **(K)**, and Rembrandt **(L)**. **(M)** Bar plot summarizing C-indices of the RLPI in 6 LGG cohorts. Data are presented as mean ± 95% CI. AUC, area under the receiver operating characteristic curve.

We then conducted univariate Cox regression analyses in the TCGA-LGG training cohort and 5 external validating cohorts. Results showed that RLPI as a continuous variable was significantly associated with a shorter time to survival in all datasets (TCGA-LGG: HR: 3.630, 95%CI: 2.990-4.406; CCGA-LGG: HR: 2.751, 95%CI: 2.365-3.199; Gravendeel: HR: 2.363, 95%CI: 1.673-3.338; Gorovets: HR: 7.983, 95%CI: 2.510-25.388; Kamoun: HR: 2.041, 95%CI: 1.433-2.906; Rembrandt: HR: 2.475, 95%CI: 1.906-3.215, **Figures 3A–F**). In addition, we tested whether a fixed RLPI threshold could be used to classify all LGG patients as high- or low-risk. To this end, we used the “SurvivalROC” R package and found that a cutoff point of 0.137 in TCGA-LGG was able to divide all patients into high- and low-risk groups with significant differences in time to overall survival for all datasets, as shown by the Kaplan-Meier analyses (all log-rank P <  = 0.01, **Figures 3A–F**).

Next, we interrogated the predictive values of RLPI using 1-year AUC, 3-year AUC, 5-year AUC, and C-index. The 1-year, 3-year, and 5-year AUC values were 0.890, 0.883, 0.806 for TCGA-LGG, 0.787, 0.769, 0.762 for CGGA-LGG, 0.908, 0.776, 0.681 for Gravendeel, 0.709, 0.743, 0.631 for Kamoun, 0.651, 0.790, 0.764 for Rembrandt, and 0.828, 0.769, 0.657 for Gorovets (**Figures 3G–L**). The C-index values were 0.846 for TCGA-LGG, 0.725 for CGGA-LGG, 0.676 for Gravendeel, 0.684 for Rembrandt, 0.714 for Kamoun, and 0.768 for Gorovets (**Figure 3M**).

**Figure 4:**
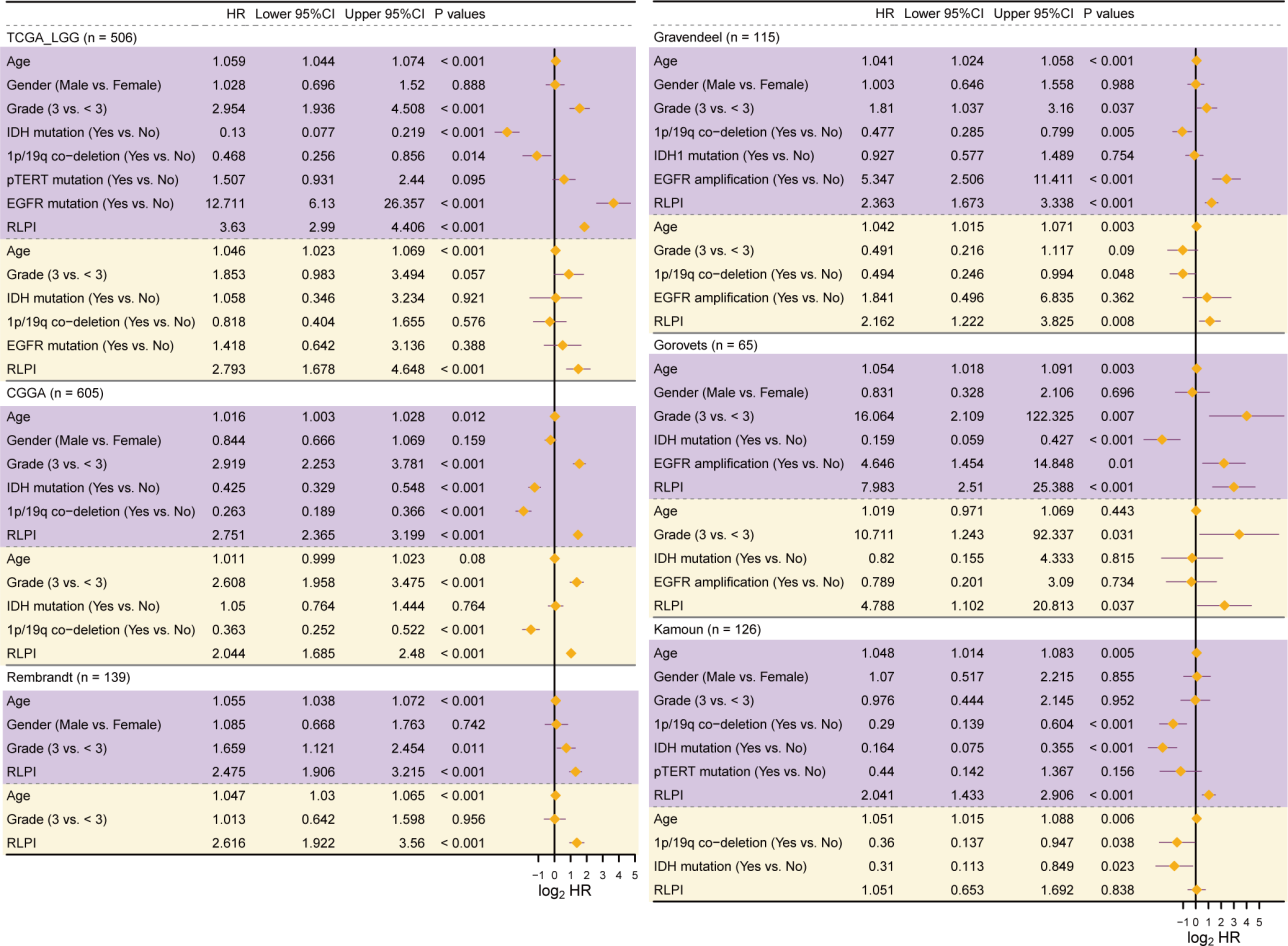
Independent validation of the R-loop prognostic index in multiple LGG cohorts. Forest plots showing hazard ratio (HR) at 95% confidence interval (CI) and the corresponding P values of RLPI, clinicopathological characteristics, and genetic alterations using both the univariate (purple area above the dashed lines) and the multivariate Cox regression analyses (yellow area below the dashed lines) in 6 LGG cohorts. Only variables with a P value < 0.05 in univariate analyses were included in multivariate analyses.

Additionally, after adjusting for common clinicopathological factors and genetic alterations such as 1p/19q co-deletion and IDH mutation, RLPI remained an independent risk factor for 5 LGG cohorts except in the Kamoun cohort (TCGA-LGG: HR: 2.793, 95%CI: 1.678–4.648; CCGA-LGG: HR: 2.044, 95%CI: 1.685–2.480; Gravendeel: HR: 2.162, 95%CI: 1.222–3.825; Gorovets: HR: 4.788, 95%CI: 1.102–20.813; Kamoun: HR: 1.051, 95%CI: 0.653–1.692; Rembrandt: HR: 2.616, 95%CI: 1.922–3.56, **Figure 4**). Together, these data nominated RLPI as a robust and independent predictor for LGG prognosis.

Next, we determined whether the prognostic value of RLPI observed in LGG could be extended to GBM and pan-glioma cohorts. In GBM cohorts, the C-index values were modest, including 0.547 (TCGA-GBM), 0.581 (CGGA-GBM), 0.615 (Gravendeel), 0.594 (Rembrandt), 0.596 (Murat), 0.549 (Ducray), 0.456 (Donson), and 0.486 (Paugh) (**Supplementary Figure S2A**), indicating limited predictive ability in GBM. Time-dependent ROC analyses showed overall unsatisfying AUC values across 1-, 3-, and 5-year survival endpoints (**Supplementary Figure S2B**), further suggesting that RLPI does not consistently perform well in GBM. In contrast, RLPI demonstrated stronger prognostic performance in pan-glioma cohorts. The C-index values were 0.840 (TCGA), 0.734 (CGGA), 0.704 (Gravendeel), and 0.673 (Rembrandt) (**Supplementary Figure S2C**). Corresponding 1-, 3-, and 5-year AUC values were consistently high across datasets (**Supplementary Figure S2D**). Collectively, these results suggest that RLPI shows limited prognostic utility in GBM alone but performs robustly in LGG and pan-glioma settings, supporting a relatively LGG-specific predictive advantage of the model.

### 3.5 RLPI converges with a mesenchymal-like state in LGG cells

**Figure 5:**
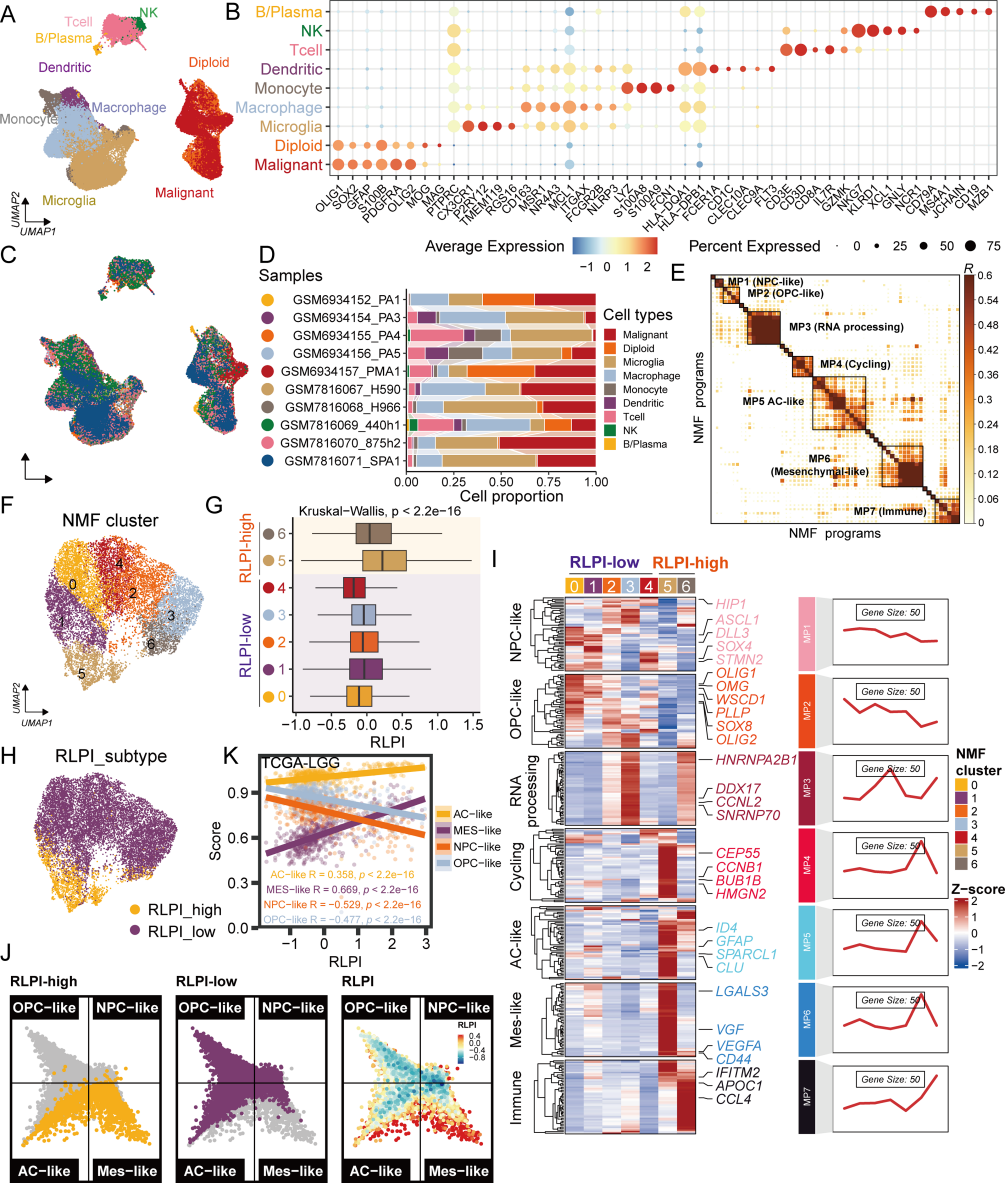
RLPI-high glioma cells were associated with a mesenchymal-like subtype. **(A)** UMAP plot showing 9 major cell types identified from scRNA-seq data of 10 LGG samples. **(B)** Dot plot displaying the expression levels of canonical marker genes for each major cell type shown in **(A)**. Dot size represents the percentage of cells expressing a given gene, while color intensity reflects expression levels. **(C)** UMAP plot showing cell-type distributions colored by individual LGG samples. **(D)** Percentages of the 9 major cell types across the 10 LGG samples. **(E)** Heatmap displaying pairwise correlation clustering of intratumoral gene expression programs (GEPs). GEPs were derived using non-negative matrix factorization (NMF) and grouped into seven consensus metaprograms (MPs). **(F)** UMAP plot of glioma cells clustered based on 7 MPs. **(G)** RLPI scores among NMF clusters of glioma cells. **(H)** UMAP plot of RLPI subtypes in glioma cells. **(I)** Heatmap of mRNA expression of signature genes from 7 MPs across glioma NMF clusters. **(J)** Two-dimensional butterfly plot visualizing molecular subtype signature scores as defined by Neftel et al. Each quadrant represents a subtype—mesenchymal-like (Mes-like), neural progenitor-like (NPC-like), astrocyte-like (AC-like), and oligodendrocyte progenitor-like (OPC-like). The position of each cell reflects its relative subtype signature scores; colors indicate RLPI subtypes or RLPI levels. **(K)** Correlations between RLPI and the four molecular subtype signature scores (Mes-like, NPC-like, AC-like, and OPC-like) in the TCGA-LGG cohort. Colors indicate the corresponding molecular signatures.

To decipher the biological underpinnings of RLPI at the single-cell level, we first collected 2 scRNA-seq datasets, which contain 10 LGG samples (**Supplementary Figure S3A**). After quality control, a total of 54859 cells were analyzed by unsupervised clustering. Individual cells were identified as either cancer or normal based on inferred copy number alterations using the CopyKat algorithm (**Supplementary Figure S4**). By combining CopyKat analysis and marker gene expression, each cluster was classified as either malignant cells (OLIG1, SOX2, GFAP, and PDGFRA), microglial cells (P2RY12 and TMEM119), macrophages (CD163, MSR1, and MCL1), monocytes (LYZ, S100A8, and S100A9), dendritic cells (HLA-DQA1 and FCER1A), T cells (CD3D and CD3E), natural killer cells (NKG7, KLRD1 and XCL1), or B/Plasma cells (CD79A and CD19) (**Figures 5A, B**). As expected, glioma and myeloid cells are the two most abundant cell types in LGG, followed by T cells (**Figures 5C, D**).

Next, cNMF was utilized to analyze transcriptional heterogeneity of malignant cells. This approach yielded seven distinct consensus meta-programs (designated MP1 to MP7), each reflecting shared patterns of intratumoral heterogeneity observed across the analyzed samples (**Figure 5E****;****Supplementary Table S6**). Notably, four of these meta-programs corresponded to previously reported glioblastoma cellular states characterized by signature genes indicative of neural progenitor cell-like (NPC-like, MP1), oligodendrocyte progenitor cell-like (OPC-like, MP2), astrocyte-like (AC-like, MP5), and mesenchymal-like (Mes-like, MP6) states, respectively. Additionally, MP7 (Immune) was enriched for immune-related genes such as IFITM2, APOC1, and HLA-DRA, suggesting an immune-associated cellular state (**Figure 5E**). Using these meta-programs, we performed unsupervised clustering, which delineated seven distinct glioma cell clusters (**Figure 5F**; **Supplementary Figure S3B**). Clusters 5 and 6 exhibited higher RLPI (**Figure 5G**) and were therefore designated as RLPI-high clusters (**Figure 5H**). These RLPI-high clusters displayed increased expression of signature genes from MP4-MP7, indicative of a more aggressive cellular state associated with proliferative, AC-like, Mes-like, and immune features (**Figure 5I**; **Supplementary Figure S3C**). We further projected all glioma cells onto a two-dimensional butterfly plot, with each quadrant representing a distinct subtype state. Notably, most RLPI-high cells were localized within the Mes-like quadrant (**Figure 5J**). RLPI was positively correlated with the Mes-like score (R = 0.347, P < 0.001) and negatively correlated with OPC-like (R = -0.281, P < 0.001) and NPC-like scores (R = -0.252, P < 0.001) (**Supplementary Figure S3D**). Similar correlation patterns were observed in both the TCGA-LGG and CGGA-LGG datasets (**Figure 5K**; **Supplementary Figure S3E**). Taken together, our integrative analyses implicate a role of RLPI in promoting or sustaining the Mes-like state in glioma cells.

### 3.6 RLPI is associated with active oncogenic signaling pathways and metabolism

**Figure 6:**
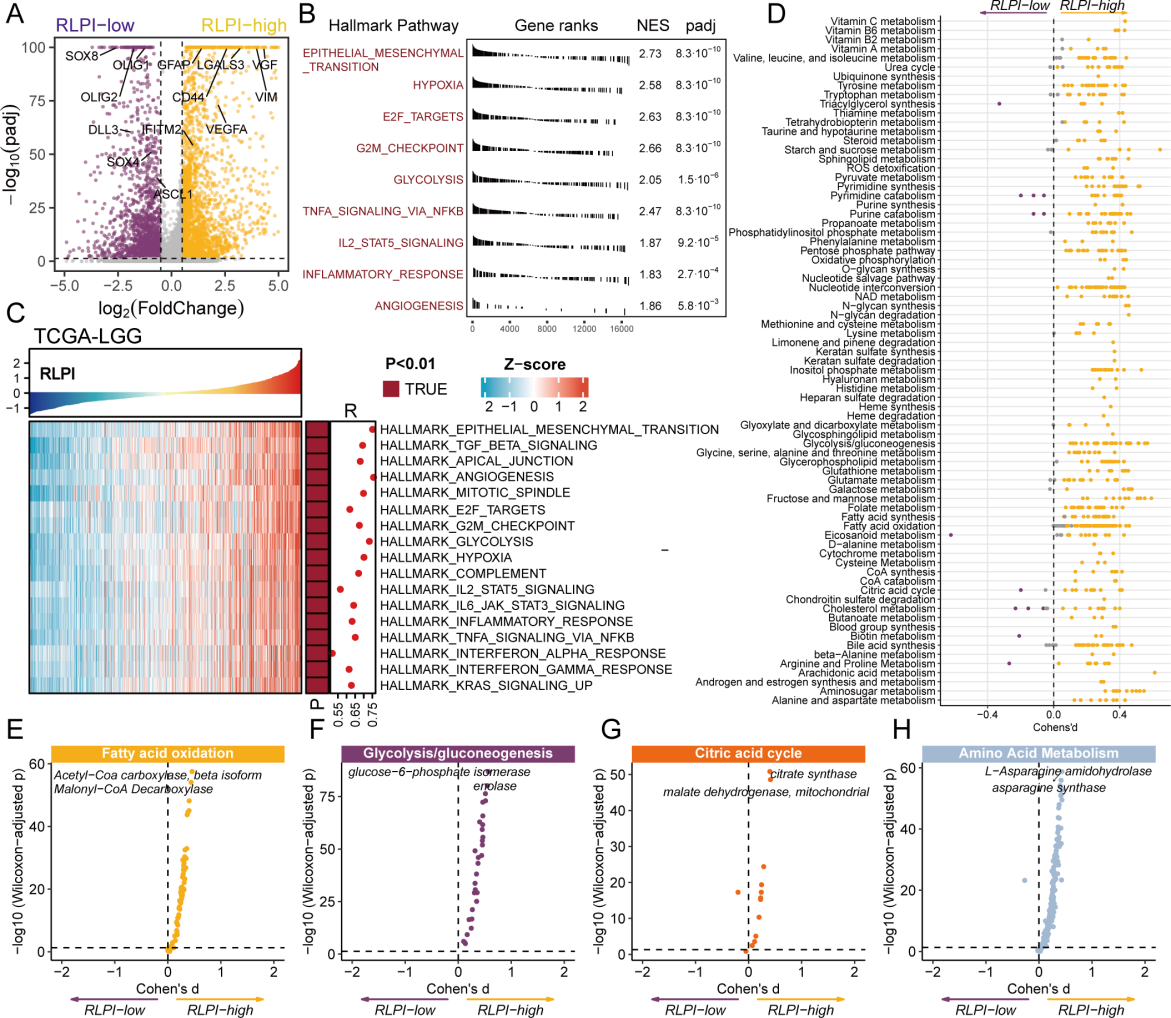
Biological characteristics of RLPI-defined subtypes in glioma cells. **(A)** Vocalnol plot showing differentially expressed genes between RLPI-high and RLPI-low glioma cells. Yellow dots indicate up-regulated genes, whereas purple dots indicate down-regulated genes. **(B)** Gene set enrichment analysis (GSEA) comparing RLPI-high versus RLPI-low glioma cells. **(C)** Associations of the RLPI with Hallmark pathway activities in the TCGA-LGG cohort. The upper panel displays RLPI scores sorted in ascending order. The lower panel shows z-scores for Hallmark pathway activity derived from single-sample GSEA (ssGSEA). Pearson’s correlation coefficients and corresponding P-values are annotated on the left side of the heatmap. **(D)** Differential activity of metabolic reactions between RLPI-high and RLPI-low glioma cells. Each dot represents a metabolic reaction, grouped by Recon2 metabolic pathways and colored according to the sign of Cohen’s d statistic. **(E–H)** Differential Compass scores between RLPI-high and RLPI-low cells for key metabolic processes, including fatty acid oxidation **(E)**, glycolysis/gluconeogenesis **(F)**, citric acid cycle **(G)**, and amino acid metabolism **(H)**. NES, normalized enrichment scores.

To gain molecular insights into RLPI-high/low clusters, we performed GSEA on clusters with high and low RLPI using the result of differential gene analysis (**Figure 6A****;****Supplementary Table S7**). RLPI-high clusters were significantly enriched in Hallmark pathways, including epithelial-mesenchymal transition (EMT), hypoxia, glycolysis, angiogenesis, cell cycle, and inflammatory response (NES > 1.8, FDR <0.05) (**Figure 6B**). Similar trends were found in the TCGA-LGG (**Figure 6C**) and CGGA-LGG datasets (**Supplementary Figure S5**).

Considering metabolism reprogramming is an established regulator of tumor progression, we investigated metabolism states of RLPI clusters using Compass, an algorithm for single-cell flux balance analysis. Our analyses showed that RLPI-high clusters were significantly more metabolically active (**Figure 6D****;****Supplementary Table S8**). Notably, most reactions from classical metabolism pathways such as fatty acid oxidation, glycolysis, tricarboxylic acid cycle (TCA), and amino acid metabolism were more active in RLPI-high clusters (**Figures 6E–H**), highlighting an increased demand for energy and biomass production.

### 3.7 RLPI reshapes the intercellular interactions in the tumor microenvironment

**Figure 7:**
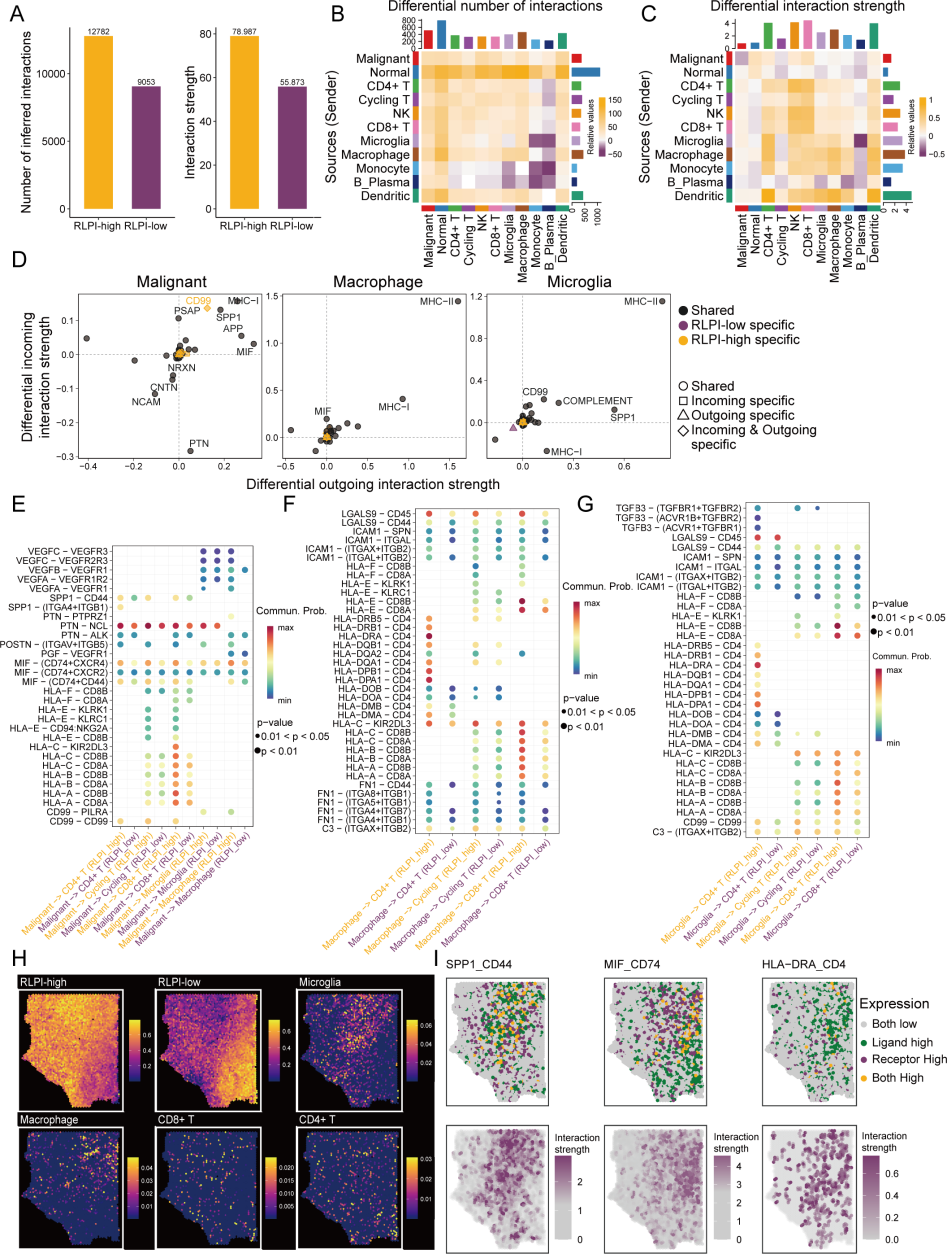
RLPI-high LGG exhibited enhanced intercellular communications. **(A)** Bar plots comparing the total number and strength of inferred cellular interactions between RLPI-high and RLPI-low LGG tumors. **(B)** Differential number of interactions among major cell types. Yellow indicates a higher number of interactions in RLPI-high tumors, while purple indicates more interactions in RLPI-low tumors. **(C)** Differential strength of interactions among major cell types. Yellow indicates stronger interactions in RLPI-high tumors, while purple indicates stronger interactions in RLPI-low tumors. **(D)** Differential incoming and outgoing interaction strength in malignant, macrophage, and microglia cells between RLPI-high and RLPI-low tumors. **(E–G)** Dot plots comparing the ligand-receptor (LR) pairs between cell types in RLPI-high versus RLPI-low tumors: glioma–immune cell interactions **(E)**, macrophage-T cell interactions **(F)**, and microglia-T cell interactions **(G)**. Dot size represents the P-value, and color reflects the communication probability for each LR pair. **(H)** Spatial distribution of cell type proportions in one LGG sample, estimated using RCTD. **(I)** Spatial distribution of ligand-receptor expression and interaction strength, inferred by SpaGene.

Given the critical role of intercellular interactions in modulating tumor behavior, we examined the impact of RLPI on cellular communications within LGG samples. To this end, 10 LGG samples were stratified into RLPI-high and RLPI-low groups based on the median RLPI value (**Supplementary Figure S6A**). Using the CellChat algorithm, we found that the RLPI-high group exhibited both a greater number and stronger intensity of inferred intercellular interactions compared to the RLPI-low group (**Figure 7A**). We next focused on specific ligand-receptor interactions among glioma, myeloid, and T cells, which constitute major cellular components in LGG. In the RLPI-high group, glioma and myeloid cells, acting as primary signal senders, demonstrated increased communication with CD4+ and CD8+ T cells in both frequency and interaction strength (**Figures 7B, C**). Notably, glioma cells in the RLPI-high group showed elevated outgoing interactions, via MHC-I, CD99, SPP1, APP, and MIF pathways (**Figure 7D**). Macrophage and microglia displayed similarly increased outgoing signals, including MHC-I, MHC-II, complement, ICAM1, and galectin signaling (**Figure 7D**). Enhanced HLA-A/B/C/E-CD8A/B signaling was detected from glioma and myeloid cells to CD8+ T cells, while HLA-D-CD4 interactions between myeloid cells and CD4+ T cells were also augmented in the RLPI-high group (**Figures 7E–G**; **Supplementary Figure S6B–C**). Additionally, chemokine signaling pathways, including CCL3, CCL4, CCL5, and CXCL16, were upregulated in RLPI-high LGG (**Supplementary Figure S6D**). Concurrently, immunosuppressive signaling was also elevated in RLPI-high LGG, as evidenced by increased MIF-CD74, SPP1-CD44, and TGFB3-TGFBR interactions (**Figures 7E**, **6G**). To further explore the spatial organization of these cell populations, we leveraged RCTD to map the annotated scRNA-seq dataset to the LGG ST data. This analysis revealed that RLPI-high glioma cells tended to co-localize with microglia, macrophages, and CD8+ T cells, whereas RLPI-low glioma cells were relatively excluded from these immune cell populations (**Figure 7H**). Moreover, several ligand-receptor pairs, including SPP1-CD44, MIF-74 and HLA-DRA-CD4 were predominantly localized within the RLPI-high area (**Figure 7I**), which provided spatially resolved evidence supporting enhanced immune-related cell-cell interactions in RLPI-high tumors. Collectively, these findings indicate that high RLPI levels are associated with enhanced antigen presentation and immune cell engagement, accompanied by concurrent immunosuppressive signaling.

### 3.8 RLPI is associated with high immune infiltration and ICI response

**Figure 8:**
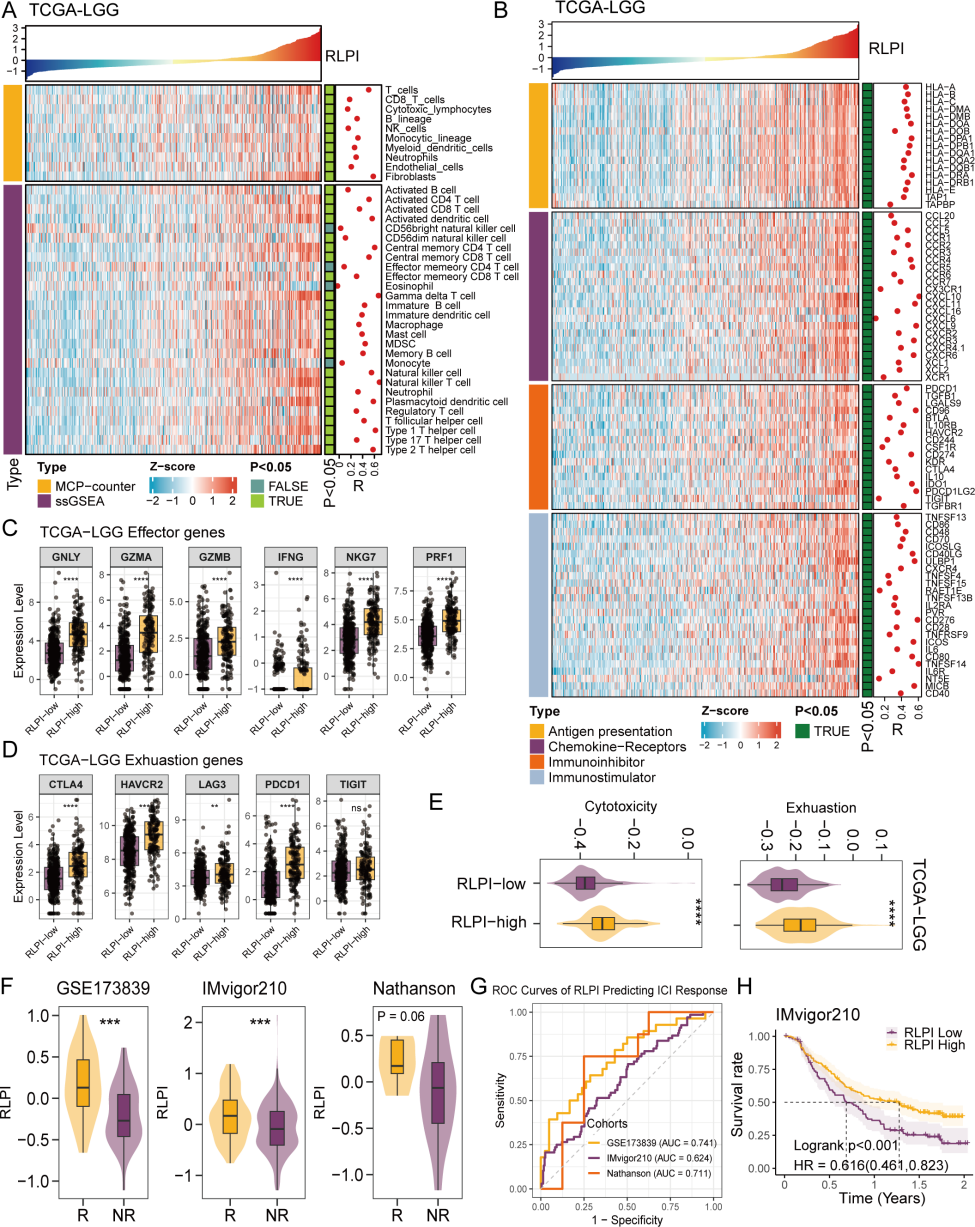
Immune-related characteristics of RLPI subtypes in LGG. **(A)** Associations of RLPI with immune cell infiltration in the TCGA-LGG cohort. The upper panel displays RLPI scores sorted in ascending order. The middle and lower panels show inferred immune cell infiltration levels derived from MCP-counter and single-sample gene set enrichment analysis (ssGSEA), respectively. Pearson’s correlation coefficients and corresponding P-values between infiltration levels and RLPI are annotated on the left side of the heatmap. **(B)** Associations between RLPI and immune-related gene expression in the TCGA-LGG cohort. The upper panel shows RLPI scores sorted in ascending order. The lower panel presents the expression of immune-related gene panels as z-scores. Pearson’s correlation coefficients and P-values are shown on the left side of the heatmap. Boxplot comparing the expression levels of CD8+ T cell cytotoxic **(C)** and exhaustion **(D)** markers between RLPI-high and RLPI-low groups in the TCGA-LGG cohort. **(E)** Bxplots comparing the CD8+ T cell cytotoxic and exhaustion scores between RLPI-high and RLPI-low groups in the TCGA-LGG cohort. **(F)** Box plots comparing RLPI scores between responders and non-responders to immune checkpoint inhibitors (ICI) in the GSE173839, IMvigor210, and Nathanson cohorts. **(G)** AUC curve analyses of RLPI for predicting ICI response in the immunotherapy cohorts. **(H)** Kaplan-Meier analysis showing overall survival in patients with urothelial carcinoma treated with atezolizumab, stratified by RLPI-high (yellow) and RLPI-low (purple) subtypes. ns, not significant; **P < 0.01; ***P < 0.001; ****P < 0.0001.

Considering the close association between RLPI and the cellular communication landscape, we next explored its potential link to immune infiltration. Using MCP-Counter and ssGSEA algorithms, we inferred the abundance of various immune cell populations across TCGA-LGG samples. Elevated RLPI was significantly correlated with increased infiltration of immune effector cells, including activated CD4+ (R = 0.509, P < 0.001) and CD8+ T cells (R = 0.346, P < 0.001), as well as immunosuppressive populations such as regulatory T cells (R = 0.293, P < 0.001) and Myeloid-derived suppressor cells (R = 0.443, P < 0.001) (**Figure 8A**). We further examined the relationship between RLPI and immune-related gene expression. Higher RLPI was significantly correlated with increased expression of genes involved in antigen presentation (e.g., HLA-A, HLA-DRA), chemokine/receptors (e.g., CCL5, CCR1), immunostimulatory molecules (e.g., TNFSF13, CD86), and immune checkpoint molecules (e.g., PDCD1, CD274, HAVCR2) (**Figure 8B**). These associations were consistently observed in the CGGA-LGG cohort (**Supplementary Figures S7A, B**). Also, RLPI-high tumors exhibited elevated expression of CD8+ T-cell effector and exhaustion markers (**Figures 8C–E**; **Supplementary Figures S7C, D**). Altogether, these findings suggest that high RLPI is associated with enhanced immune infiltration and concurrent immunosuppressive signaling, aligning with the intercellular communication pattern observed in RLPI-high LGG.

To assess the potential clinical relevance of RLPI in the context of immunotherapy, we analyzed 5 independent ICI-treated cohorts. Notably, RLPI was significantly higher in patients who exhibited favorable responses to ICI (**Figure 8F**; **Supplementary Figures S7E, F**). Higher RLPI was predictive of ICI response, with AUC values of 0.741 in GSE173839, 0.711 in the Nathanson cohort, 0.624 in IMvigor210, 0.632 in Nabet, and 0.752 in Finn (**Figure 8G**; **Supplementary Figures S7E, F**). Furthermore, elevated RLPI was associated with prolonged overall survival following ICI treatment in the IMvigor210 dataset (**Figure 8H**).

### 3.9 RLPI is correlated with resistance to radiotherapy and temozolomide

**Figure 9:**
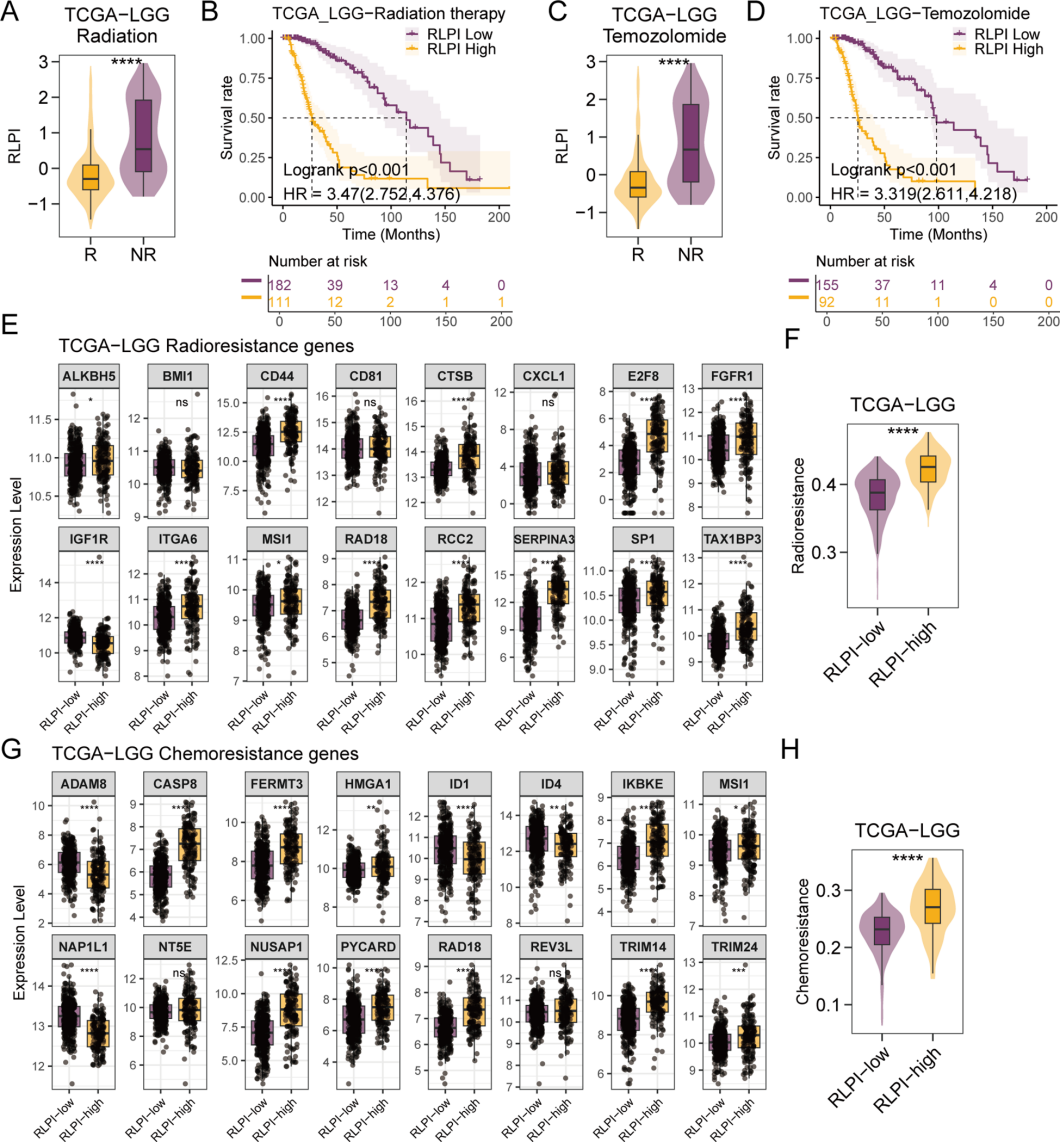
Association of RLPI with radiotherapy and temozolomide resistance in the TCGA-LGG cohort. **(A)** Boxplot comparing RLPI scores between radiation therapy responders and non-responders in the TCGA-LGG cohort. **(B)** Kaplan-Meier survival curves comparing overall survival between RLPI-high and RLPI-low groups among patients receiving radiation therapy in the TCGA-LGG cohort. P values were derived from log-rank tests. The hazard ratio (HR) and 95% confidence interval (CI) were calculated using univariate Cox regression analysis. **(C)** Boxplot comparing RLPI scores between temozolomide responders and non-responders in the TCGA-LGG cohort. **(D)** Kaplan-Meier survival curves comparing overall survival between RLPI-high and RLPI-low groups among patients treated with temozolomide in the TCGA-LGG cohort. **(E)** Boxplot comparing the expression levels of radiotherapy resistance-related genes between RLPI-high and RLPI-low groups in the TCGA-LGG cohort. **(F)** Boxplot comparing the radiotherapy resistance-related gene signature scores between RLPI-high and RLPI-low groups in the TCGA-LGG cohort. **(G)** Boxplot comparing the expression levels of temozolomide resistance-related genes between RLPI-high and RLPI-low groups in the TCGA-LGG cohort. **(H)** Boxplot comparing the temozolomide resistance-related gene signature scores between RLPI-high and RLPI-low groups in the TCGA-LGG cohort. ns, not significant; *P < 0.05; **P < 0.01; ***P < 0.001; ****P < 0.0001.

Next, we explored the association of RLPI with response to radiotherapy (RT) and temozolomide treatment, which are conventional treatment for LGG. Among patients receiving RT, non-responders exhibited significantly higher RLPI scores compared with responders (**Figure 9A**). Moreover, RLPI-high patients had markedly shorter overall survival than RLPI-low patients within the RT-treated subgroup (**Figure 9B**). Consistent findings were observed in patients treated with temozolomide (**Figures 9C, D**), indicating a strong association between elevated RLPI and poor therapeutic response. To further substantiate this observation, we curated experimentally validated radioresistance- and temozolomide-resistance-related genes in glioma. Most resistance-associated genes were more highly expressed in the RLPI-high group (**Figures 9E**, **G**). In addition, single-sample gene set enrichment (ssGSEA) analysis was performed to generate RT and temozolomide resistance scores based on these gene sets. RLPI-high tumors exhibited significantly higher resistance scores (**Figures 9F**, **H**). Similar results were validated in the CGGA-LGG cohort (**Supplementary Figure S8**). Collectively, these findings demonstrated that elevated RLPI correlated with resistance to RT and temozolomide in LGG.

### 3.10 Impact of key RLPI genes on glioma cell proliferation and invasion

**Figure 10:**
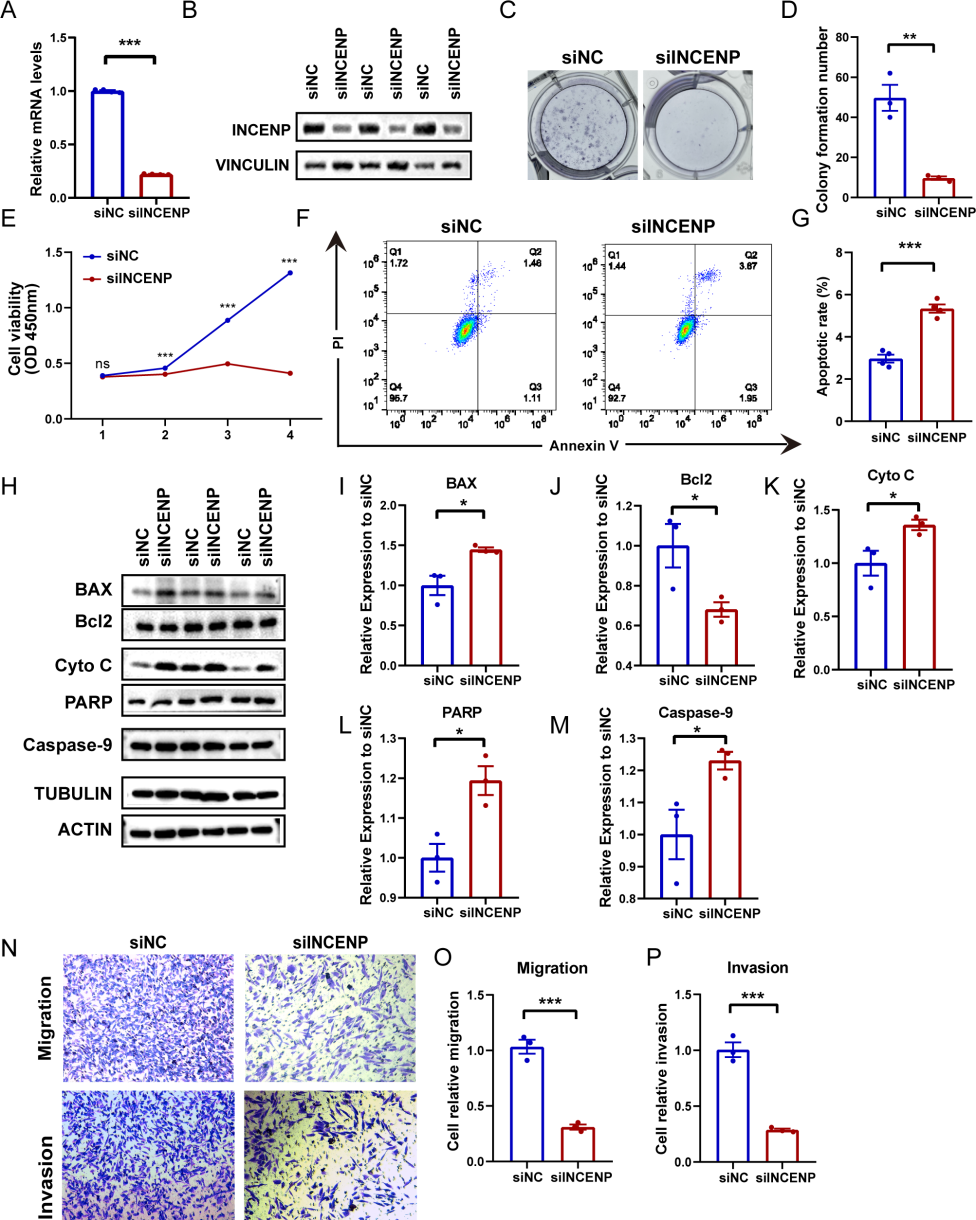
Effects of INCENP knockdown on the proliferation, apoptosis, and metastasis of Hs683 cells. **(A, B)** Validation of siRNA-mediated INCENP knockdown efficiency in Hs683 cells using qPCR **(A)** and Western blotting **(B)**. **(C)** Representative images of colony formation assays in cells transfected with siNC or siINCENP. **(D)** Quantification of the colony numbers shown in **(C)** (mean ± SEM, n = 3). **(E)** Cell viability assessed by the CCK-8 assay (mean ± SEM, n = 6). **(F)** Flow cytometric analysis of apoptosis in the siNC and siINCENP groups. **(G)** Quantification of the apoptotic cell rate (mean ± SEM, n = 4). **(H)** Western blot analysis of apoptosis-related protein expression. **(I–M)** Densitometric quantification of the protein levels shown in **(H)**. **(N)** Representative images of Transwell assays showing cell migration and invasion. **(O, P)** Quantification of migrated and invaded cells shown in **(N)** (mean ± SEM, n = 3). *P < 0.05; **P < 0.01; ***P < 0.001.

To functionally validate our model, we selected INCENP and NCAPG, representative genes from the RLPI, based on their relatively high model weights and limited prior investigation in LGG. Elevated INCENP and NCAPG mRNA expression were significantly associated with shorter overall survival in both the TCGA-LGG and CGGA-LGG cohorts (**Supplementary Figure S9**). We confirmed effective knockdown of INCENP at both the mRNA and protein levels in Hs683 and U251 glioma cells (**Figures 10A, B**; **Supplementary Figures S10A–C**). Subsequent CCK-8 and colony formation assays demonstrated that INCENP silencing markedly suppressed glioma cell proliferation (all P < 0.05, **Figures 10C–E**; **Supplementary Figures S10D–F**). Apoptotic cell death, evaluated via Annexin V and PI staining, was significantly increased following INCENP knockdown (**Figures 10F, G**; **Supplementary Figures S10G–H**). Accordingly, proteins involved in apoptotic pathways, such as BAX and Cyto C, were significantly dysregulated (**Figures 10H–M**; **Supplementary Figures S10I–N**). Additionally, Transwell assays revealed that knockdown of INCENP substantially impaired the migratory and invasive capabilities of glioma cells (**Figures 10N–P**; **Supplementary Figures S10O–Q**). Collectively, these results indicate that INCENP may facilitate LGG progression by promoting tumor cell proliferation, inhibiting apoptosis, and enhancing migration. In contrast, NCAPG knockdown inhibited migration and invasion in glioma cells but did not affect cell proliferation (**Supplementary Figure S11**).

## 4 Discussion

R-loop dysregulation has been well implicated in cancer (10); however, the prognostic and therapeutic implications of R-loop regulators in LGG remain largely unexplored. In this study, we established a robust R-loop related prognostic index (RLPI) for LGG by leveraging R-loop regulatory factors. In-depth analyses revealed that RLPI-high glioma cells converged with a mesenchymal state and enhanced metabolic activity. Furthermore, elevated RLPI was associated with more intercellular communications, increased immune cell infiltration, response to ICB therapy, and resistance to both RT and temozolomide.

The integration of machine learning (ML) into clinical oncology has revolutionized prognostic modeling and personalized medicine. Recent studies have demonstrated the robust capability of advanced ML algorithms in predicting clinical outcomes, and guiding therapeutic interventions (41–43). In this study, we adopted a two-pronged strategy to identify R-loop regulators with prognostic relevance in LGG. First, we performed univariate Cox regression analysis combined with bootstrap resampling to assess the robustness and stability of each regulator’s association with patient survival. Subsequently, we applied the Boruta algorithm, a feature selection method built upon random forest classifiers, to further refine the selection by eliminating irrelevant variables while preserving all potentially informative features (44). To construct a reliable prognostic model, we then developed an integrative machine learning pipeline that systematically benchmarked nine widely used survival algorithms within a nested CV framework. This framework effectively prevents information leakage and overfitting, yielding unbiased performance estimates that better reflect the model’s generalizability to unseen data (45). Using this framework, we ultimately selected Enet for its superior predictive stability to construct the RLPI based on the most informative R-loop regulators. By combining L1 and L2 penalties, Enet effectively handles the high dimensionality and multicollinearity inherent in transcriptomic data while minimizing overfitting (46). Beyond its methodological robustness, the resulting RLPI consistently demonstrated strong predictive performance across six independent LGG cohorts, underscoring its accuracy and potential clinical utility as a prognostic biomarker for risk stratification. Importantly, it provides actionable therapeutic guidance by identifying high-risk patients who, despite resisting conventional radiotherapy and temozolomide, may preferentially benefit from ICI.

Surprisingly, elevated RLPI levels were strongly associated with EMT, accompanied by increased expression of canonical mesenchymal markers such as CD44 and VGF. These molecular features closely mirror those of the mesenchymal-like subtype of GBM, which is widely recognized as the most aggressive form of the disease (36). Notably, glioma cells with high RLPI also exhibited enhanced metabolic activity, including upregulated glycolysis, fatty acid oxidation, and TCA cycle processes. It is well established that glioma cells undergo rapid glucose uptake and exhibit elevated glycolysis and oxidative phosphorylation during aggressive tumor progression (47). Heightened fatty acid oxidation and TCA cycle activity further promote the generation of acetyl-CoA, a central metabolite crucial for energy production and biosynthesis, thereby fueling tumor growth (47, 48). In line with this, acetyl-CoA carboxylase, a key enzyme in fatty acid metabolism, increased in RLPI-high cells, has been reported to promote glioma cell proliferation (49). Additionally, we observed increased activity of asparagine synthetase (ASNS), the rate-limiting enzyme for asparagine biosynthesis, in RLPI-high glioma cells. The ASNS gene resides on the long arm of chromosome 7, a region frequently amplified in GBM and associated with poor clinical outcomes (50). Elevated ASNS expression enhances glioma cell proliferation and invasiveness (50), potentially facilitating a mesenchymal-like phenotypic transition. Taken together, our findings suggest that high RLPI may contribute to LGG progression by promoting or maintaining a mesenchymal-like cellular state, supported by reprogrammed metabolic activity.

Conventional treatments for LGG, including surgical resection and chemotherapy, often carry a significant risk of recurrence (4). ICB has emerged as a promising therapeutic strategy across various cancer types, including GBM (51, 52). Consequently, identifying LGG patients who are most likely to benefit from ICB holds substantial clinical relevance. We found that elevated RLPI levels were strongly associated with enhanced intercellular communication within the TME, particularly involving pathways related to antigen presentation and immunosuppressive signaling. This pattern suggests a dysfunctional immune contexture characterized by initial immune activation followed by T cell exhaustion in RLPI-high LGG. Notably, RLPI-high patients exhibited significantly improved responses to ICB treatment, highlighting RLPI as a potential biomarker for selecting LGG patients who benefit from ICB therapy.

Many genes in the RLPI have been implicated in tumor progression, thereby reinforcing the reliability of our bioinformatic analyses. For example, HSPB1 activates G6PD to protect glioma cells from oxidative stress and promote cell proliferation (53), while IGF2BP3 suppresses ferroptosis by modulating GPX4 expression (54). SMC4 has been shown to enhance glioma cell proliferation and invasion via activation of the TGF-β/Smad signaling pathway (55). Similarly, APOBEC3C and MSN1 have been reported to drive glioma aggressiveness (56, 57). Among the top-ranked genes in the RLPI, INCENP was identified by our study as a contributor to glioma cell proliferation and invasion. INCENP encodes the inner centromere protein, a key component of the chromosomal passenger complex (CPC) (58). CPC is essential for proper chromosome alignment, segregation, and cytokinesis during mitosis (58). Disruption of INCENP function impairs CPC activity and induces apoptosis in neuroblastoma cells (59). This altogether underscores its potential as a therapeutic target in cancer. However, the molecular mechanisms by which INCENP promotes LGG progression remain to be elucidated.

Our study is not without limitations. First, although a robust RLPI was constructed and validated for LGG prognosis, the mechanistic role of R-loops in RLPI-mediated LGG progression remained unclear. Second, the findings of this study are primarily based on retrospective cohorts and thus require validation in prospective clinical settings. Moreover, investigations are warranted to elucidate the underlying mechanisms of RLPI genes in the progression of LGG.

## 5 Conclusion

Despite the inherent challenges associated with high-throughput analyses, our study represents a significant step toward elucidating the role of R-loop regulators in shaping tumor aggressiveness and the immune landscape in LGG. In summary, the RLPI emerges as a promising biomarker for predicting both clinical prognosis and response to ICB in LGG. Further studies are warranted to assess the translational potential of RLPI in clinical settings.

## Data Availability

Publicly available datasets were analyzed in this study. This data can be found here: Transcriptomic expression data generated by the Illumina HiSeq platform and corresponding clinical information for the TCGA-LGG (n = 506) and Chinese Glioma Genome Atlas-LGG (CGGA-LGG, n = 605) cohorts were obtained from the UCSC Xena platform (https://xena.ucsc.edu/) and the CGGA (http://www.cgga.org.cn/), respectively. Additionally, four LGG microarray datasets, including Gravendeel (n = 115), Gorovets (n = 65), Kamoun (n = 126), along with the Rembrandt (n = 139), and two scRNA-seq datasets (GSE222850 and GSE244433) were collected from the Gene Expression Omnibus (GEO, https://www.ncbi.nlm.nih.gov/geo/).
