# Supplementary material for: The role of R-loop aberrations in lower-grade gliomas: prognostic, immune, and metabolic implications from multi-omics and machine learning analysis
Source: Front Immunol. 2026 Apr 17;17:1758954. doi: 10.3389/fimmu.2026.1758954 (PMC13132874; doi:10.3389/fimmu.2026.1758954)
Supplement: Supplementary file 2 [file Table2.doc]

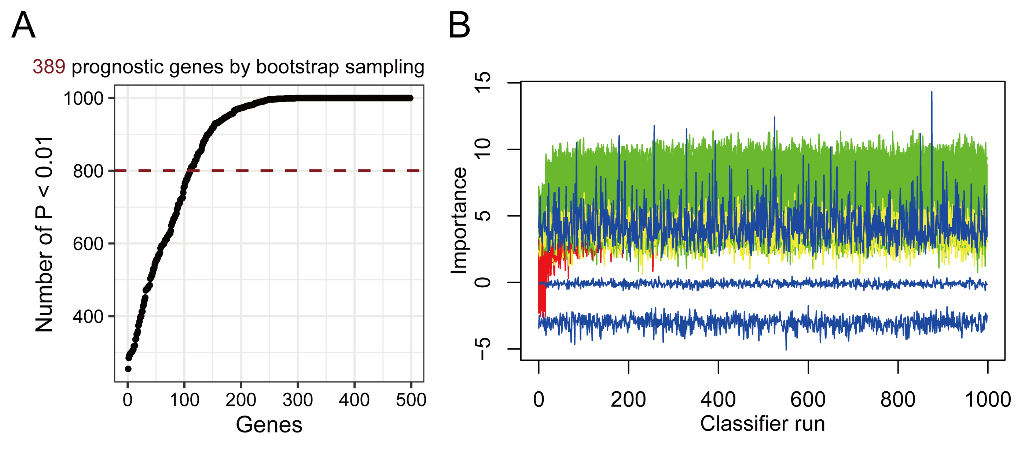


Figure S1. (A) Selection of prognostic genes robust to sample resampling. Genes achieving the P < 0.01 threshold in over 800 iterations. (B) Results of the Boruta algorithm iterations. Green indicates features considered important by the Boruta algorithm while blue represents shadow attributes.


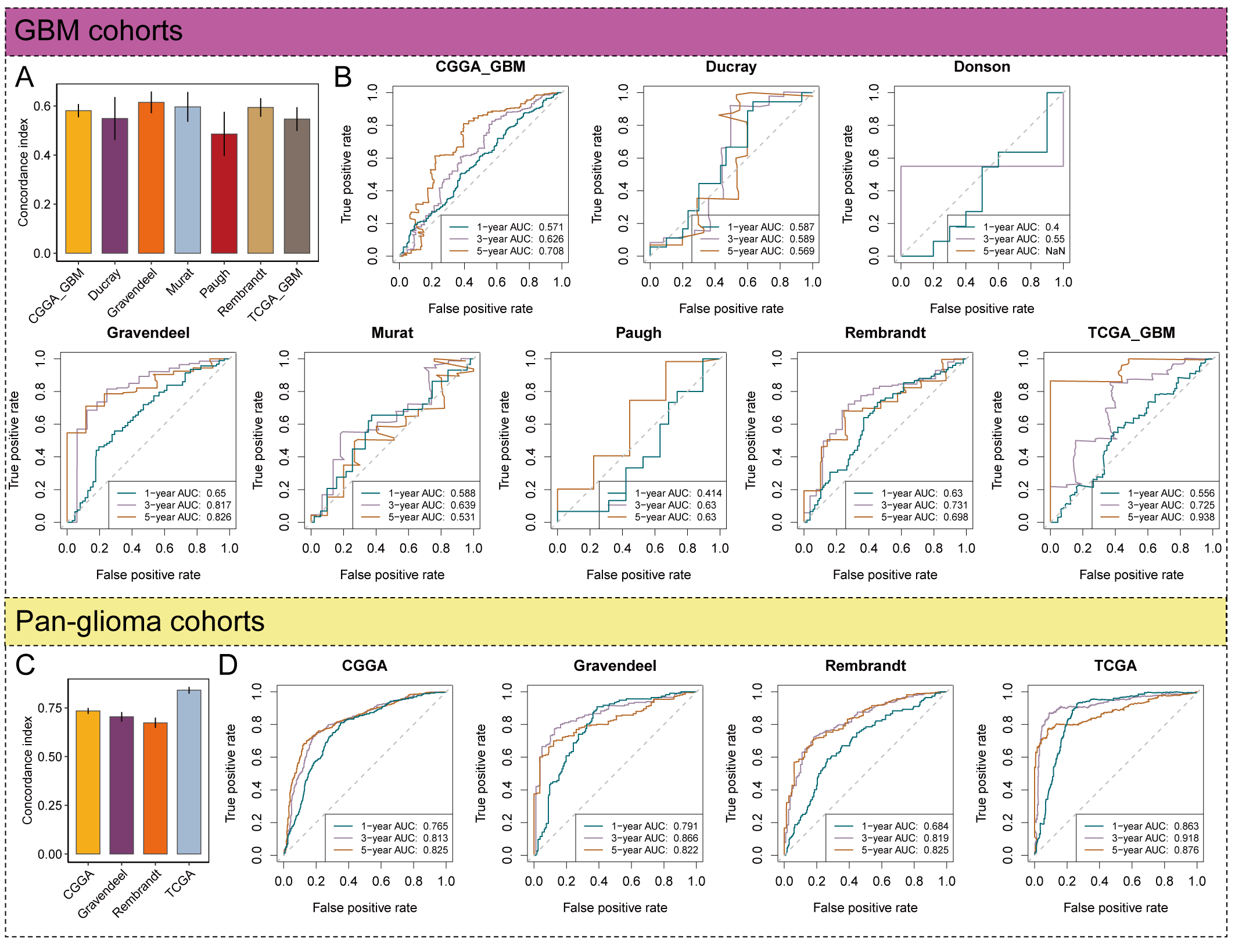


Figure S2. Evaluation of the R-loop prognostic index across glioblastoma and pan-glioma cohorts. Bar plots summarizing C-indices of the RLPI in 7 GBM (A) and 4 pan-glioma cohorts (C). Data are presented as mean ± 95% CI. Area under the receiver operating characteristic curve (AUC) analyses of RLPI for predicting prognosis at 1, 3, and 5 years in GBM cohorts from CGGA, Ducray, Donson, Gravendeel, Murat, Paugh, Rembrandt, and TCGA, and in pan-glioma cohorts from CGGA, Gravendeel, Rembrandt, and TCGA.


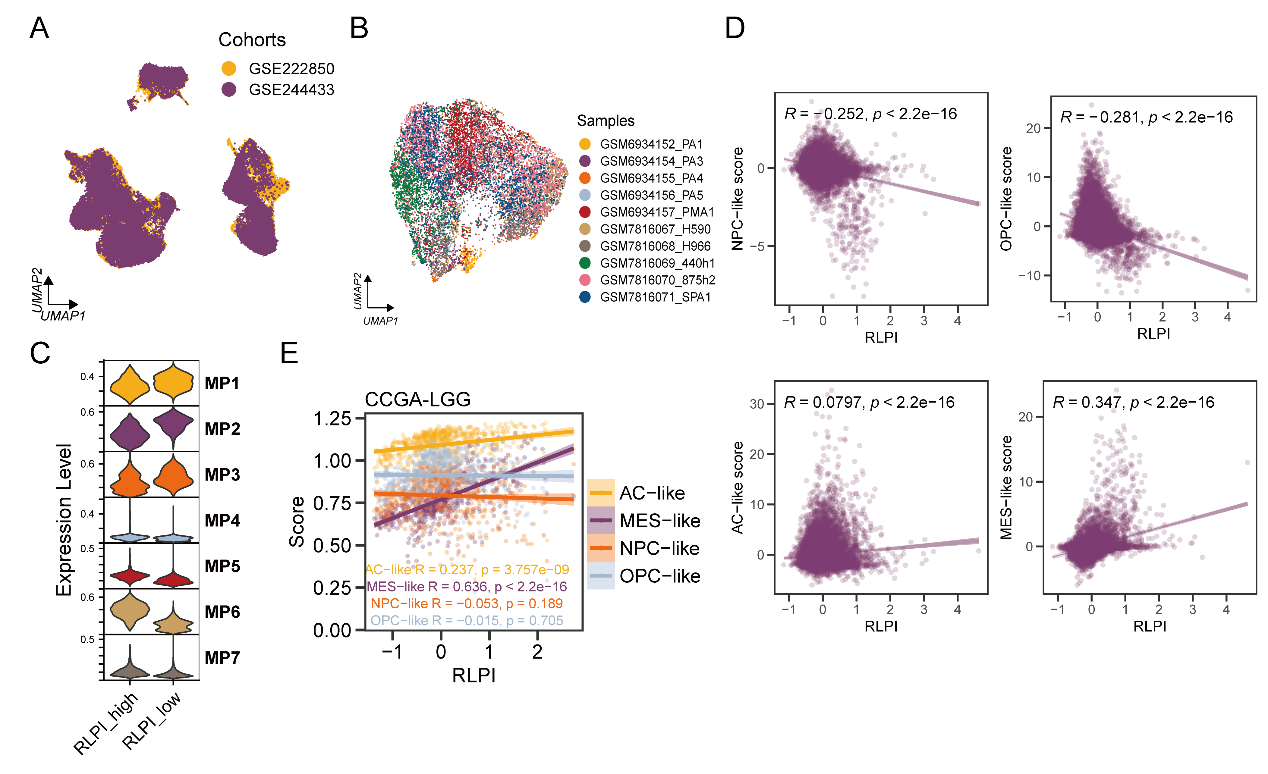


Figure S3. RLPI-high glioma cells exhibited a mesenchymal-like phenotype. (A) UMAP plot showing cell distributions colored by LGG cohorts. (B) UMAP plot showing glioma cell distributions colored by individual LGG samples. (C) Violin plot comparing the scores of 7 meta-programs between RLPI-high and RLPI-low glioma cells. (D) Correlations of RLPI with signature scores of mesenchymal-like (Mes-like), neural progenitor-like (NPC-like), astrocyte-like (AC-like), and oligodendrocyte progenitor-like (OPC-like) in glioma cells. (E) Correlations between RLPI and the four molecular subtype signature scores (Mes-like, NPC-like, AC-like, and OPC-like) in the CGGA-LGG cohort.


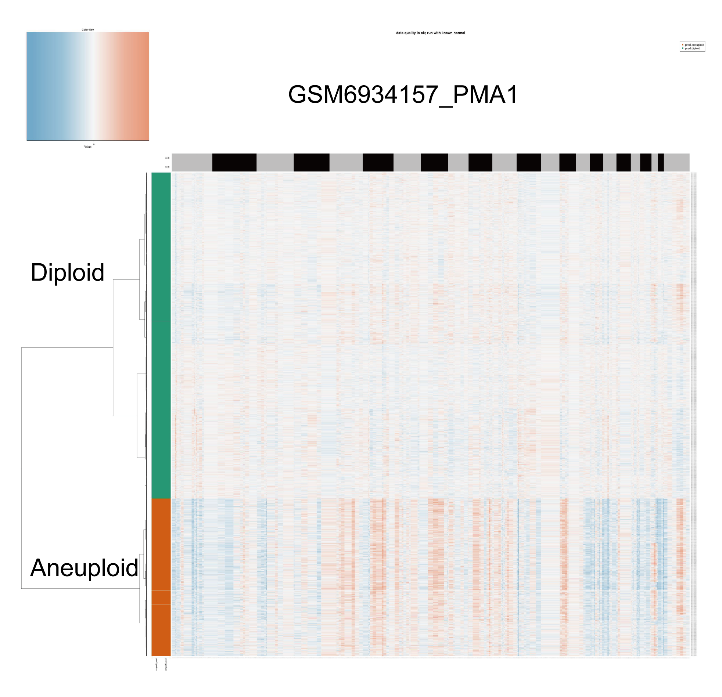


Figure S4. Heatmap illustrating inferred copy number alterations in a representative LGG single-cell RNA-sequencing sample (GSM6934157_PMA1), as determined by CopyKAT.


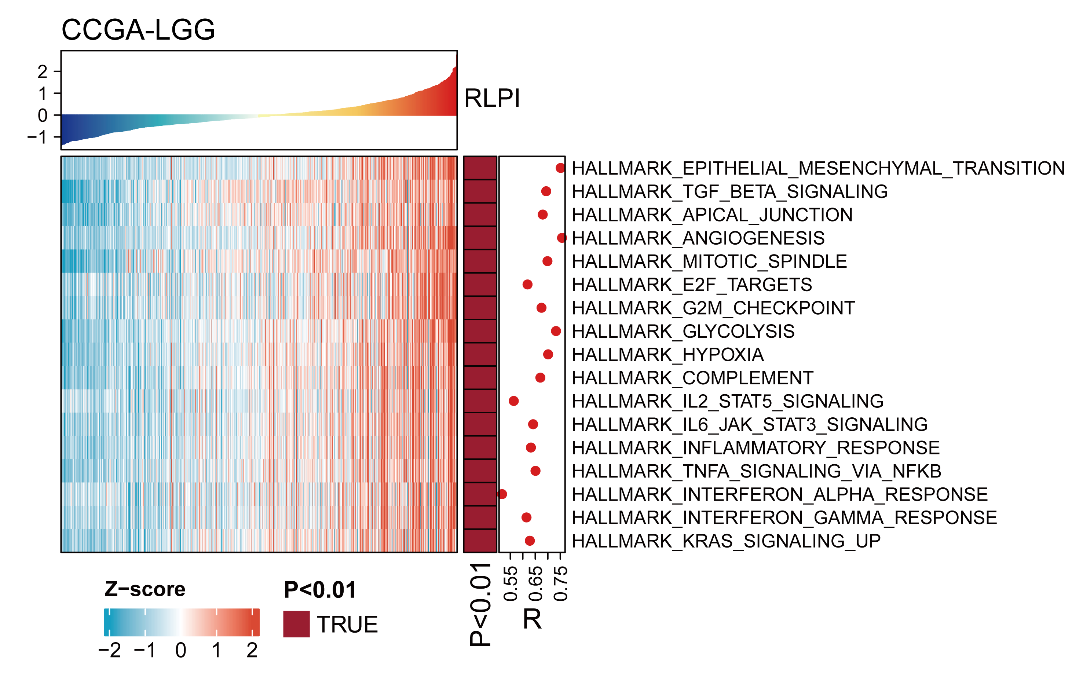


Figure S5. Associations of the RLPI with Hallmark pathway activities in the CGGA-LGG cohort. The upper panel displays RLPI scores sorted in ascending order. The lower panel shows z-scores for Hallmark pathway activity derived from single-sample gene set enrichment analysis. Pearson’s correlation coefficients and corresponding P-values are annotated on the left side of the heatmap.


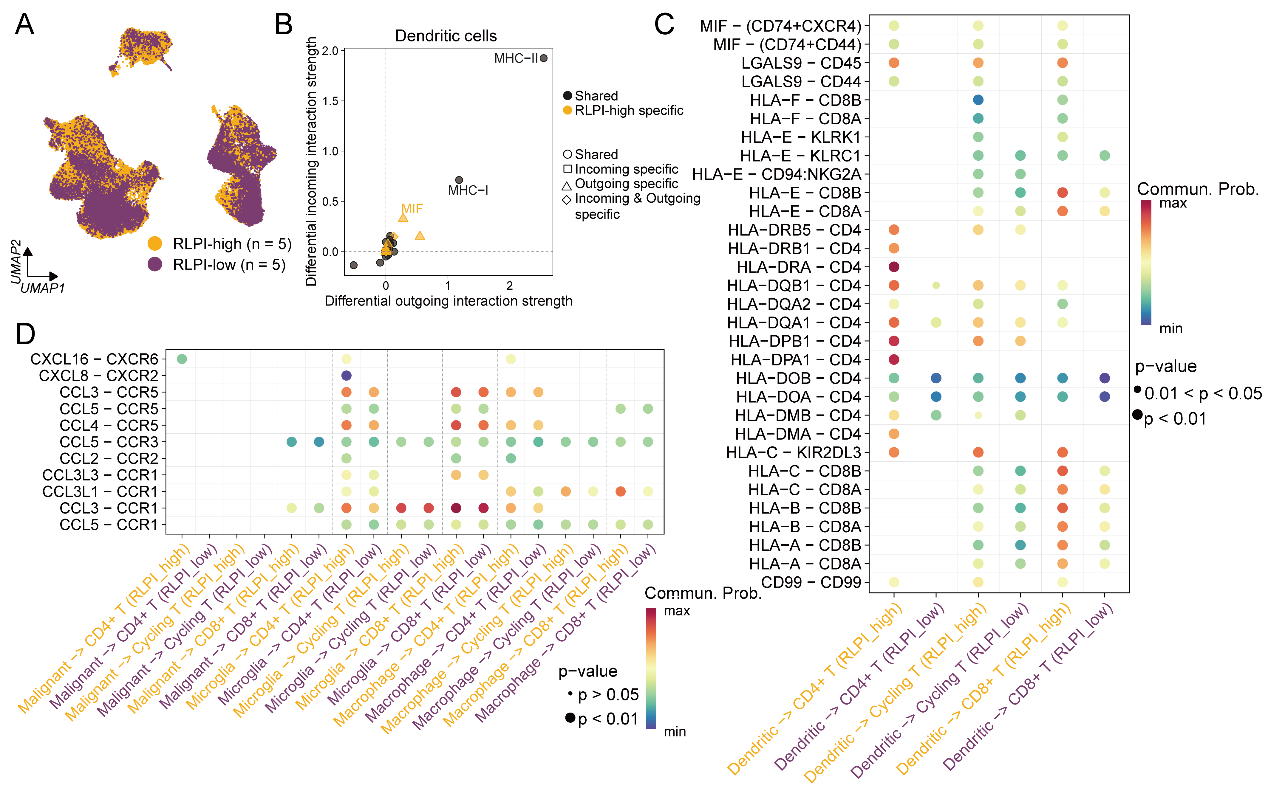


Figure S6. RLPI-high LGG exhibited enhanced intercellular communications. (A) UMAP plot showing cell distributions colored by RLPI-defined tumor groups. (B) Differential incoming and outgoing interaction strength in dendritic cells between RLPI-high and RLPI-low tumors. (C) Dot plots comparing the ligand-receptor (LR) pairs between dendritic and T cells in RLPI-high versus RLPI-low tumors. (D) Dot plots comparing the chemokine-receptor pairs in RLPI-high versus RLPI-low tumors. Dot size represents the P-value, and color reflects the communication probability for each LR pair.


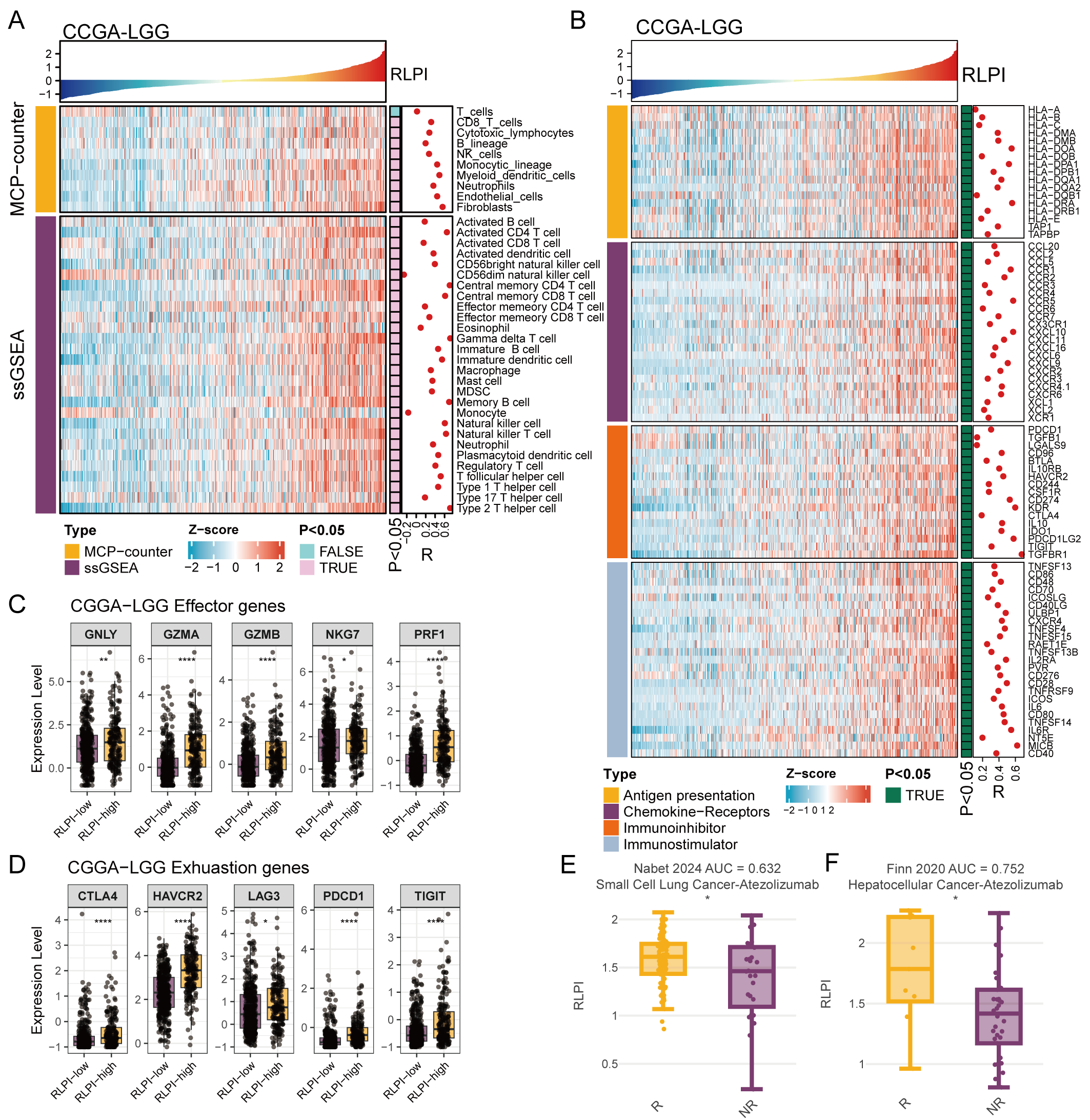


Figure S7. Immune-related characteristics of RLPI subtypes in LGG. (A) Associations of RLPI with immune cell infiltration in the CGGA-LGG cohort. The upper panel displays RLPI scores sorted in ascending order. The middle and lower panels show inferred immune cell infiltration levels derived from MCP-counter and single-sample gene set enrichment analysis (ssGSEA), respectively. Pearson’s correlation coefficients and corresponding P-values between infiltration levels and RLPI are annotated on the left side of the heatmap. (B) Associations between RLPI and immune-related gene expression in the CGGA -LGG cohort. The upper panel shows RLPI scores sorted in ascending order. The lower panel presents the expression of immune-related gene panels as z-scores. Pearson’s correlation coefficients and P-values are shown on the left side of the heatmap. Boxplots comparing the expression levels of CD8+ T cell effector markers (C) and exhaustion markers (D) between RLPI-high and RLPI-low groups in the CGGA-LGG cohort. Box plots comparing RLPI scores between responders and non-responders to anti-PD-L1 therapy in the Nabet cohort (E) and Finn (F) cohorts. *, P < 0.05; ***, P < 0.001; ****, P < 0.0001.


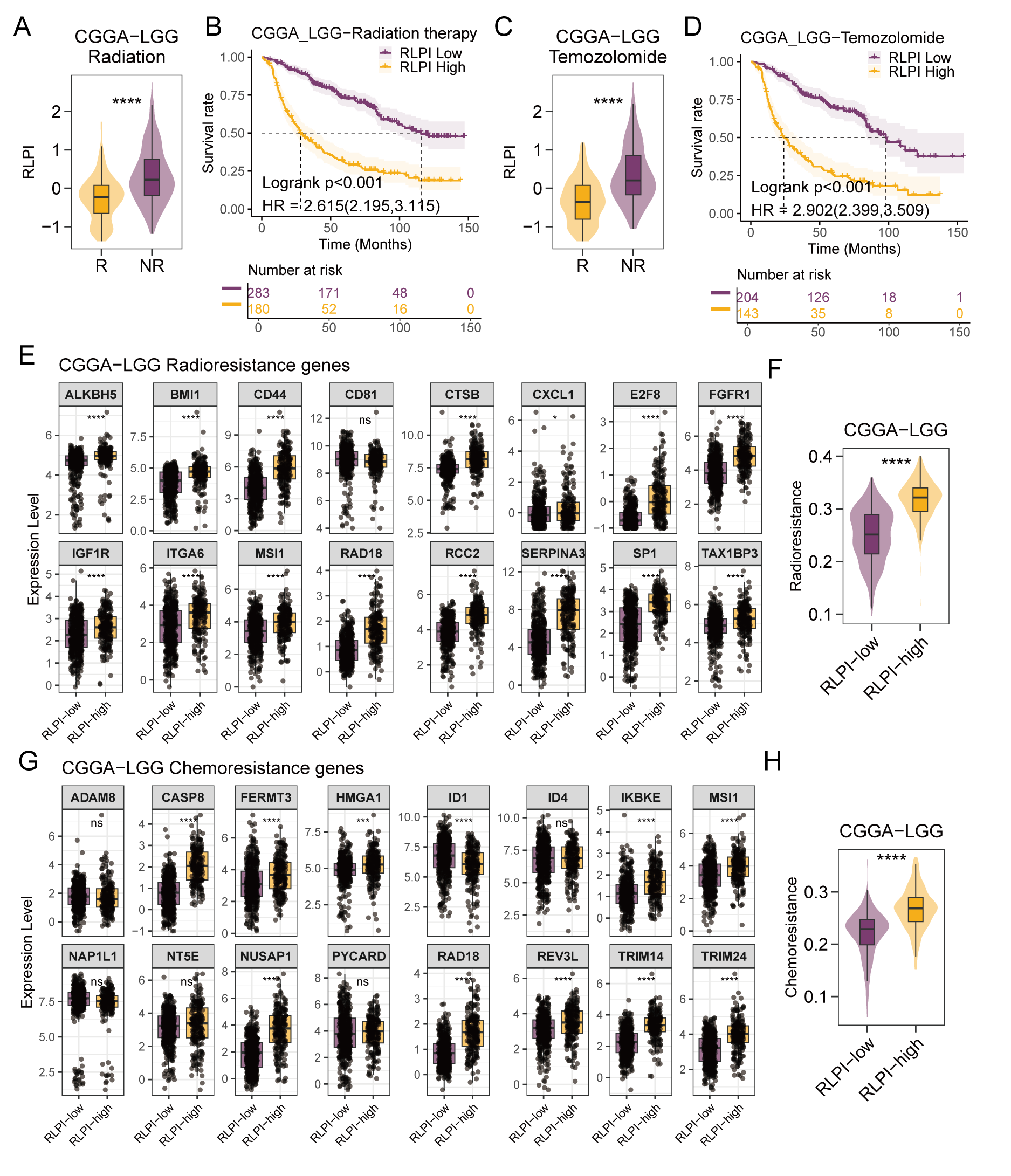


Figure S8. Association of RLPI with radiotherapy and temozolomide response in the CGGA-LGG cohort. (A) Boxplot comparing RLPI scores between radiotherapy responders and non-responders in the CGGA-LGG cohort. (B) Kaplan-Meier survival curves comparing overall survival between RLPI-high and RLPI-low groups among patients receiving radiotherapy in the CGGA-LGG cohort. P values were derived from the log-rank tests. The Hazard ratio (HR) and 95% confidence interval (CI) were computed using the univariate Cox regression analysis. (C) Boxplot comparing RLPI scores between temozolomide responders and non-responders in the CGGA-LGG cohort. (D) Kaplan-Meier survival curves comparing overall survival between RLPI-high and RLPI-low groups among patients treated with temozolomide. (E) Boxplot comparing the expression levels of radiotherapy resistance-related genes between the RLPI-high and RLPI-low groups. (F) Boxplot comparing the radiotherapy resistance–related gene signature scores between RLPI-high and RLPI-low groups. (G) Boxplot comparing the expression levels of temozolomide resistance–related genes between RLPI-high and RLPI-low groups. (H) Boxplot comparing the temozolomide resistance-related gene signature scores between RLPI-high and RLPI-low groups. ns, not significant; *, P < 0.05; ***, P < 0.001; ****, P < 0.0001.


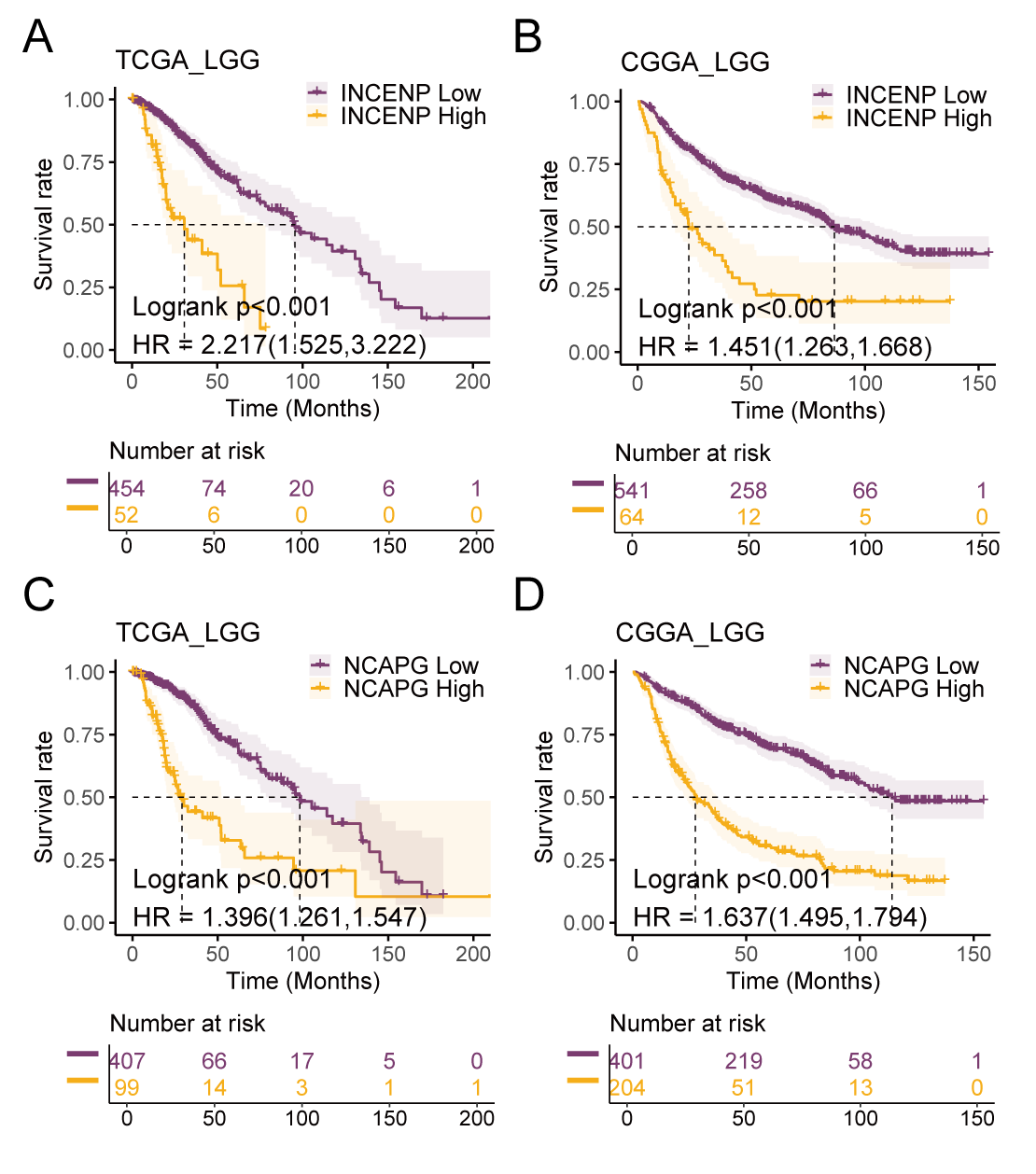


Figure S9. Kaplan-Meier curves of overall survival in INCENP-high and INCENP-low samples in TCGA-LGG (A) and CGGA-LGG (B) datasets. Kaplan-Meier curves of overall survival in NCAPG-high and NCAPG-low samples in TCGA-LGG (C) and CGGA-LGG (D) datasets. P values were derived from the log-rank tests. The Hazard ratio (HR) and 95% confidence interval (CI) were computed using the univariate Cox regression analysis.


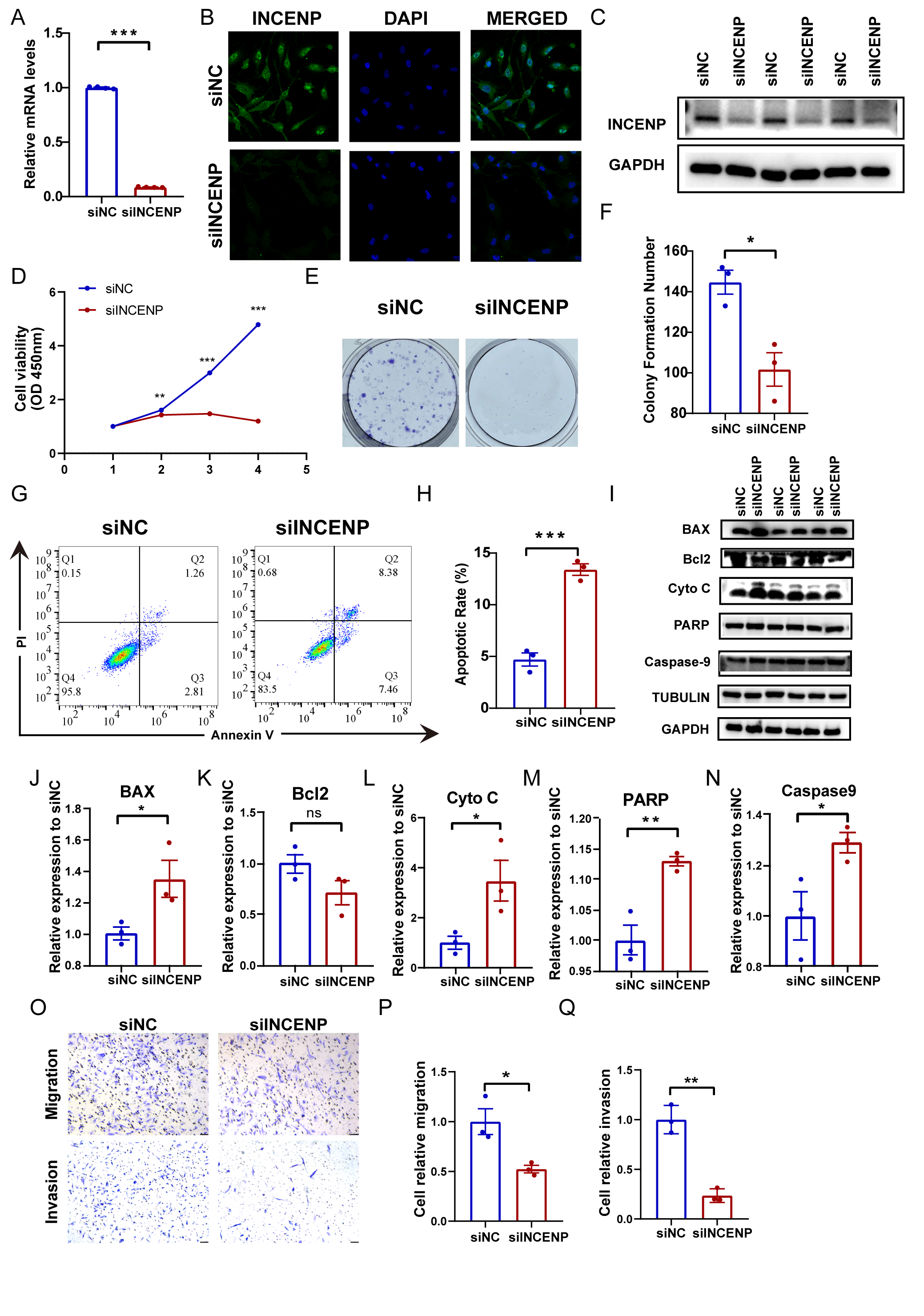


Figure S10. Effects of INCENP knockdown on the proliferation, apoptosis, and metastasis of U251 glioma cells. (A-C) Analysis of INCENP expression in U251 cells following siRNA transfection using qPCR (A), immunofluorescence staining (B), and Western blotting (C). (D) Cell viability measured by CCK-8 assay (mean±SEM, n = 6). (E) Representative images of colony formation assays. (F) Quantification of colony numbers from (E) (mean ± SEM, n = 3). (G-H) Flow cytometry assays assessing apoptosis levels (G) and subsequent quantification (H) (mean±SEM, n = 3). (I) Western blot analysis of apoptosis-related markers. (J-N) Quantification of protein expression levels from (I). (O) Representative images of Transwell migration and invasion assays. (P-Q) Quantification of migrated and invaded cells shown in (O) (mean±SEM, n = 3). ns, not significant; *, P < 0.05. **, P < 0.01; ***, P < 0.001.


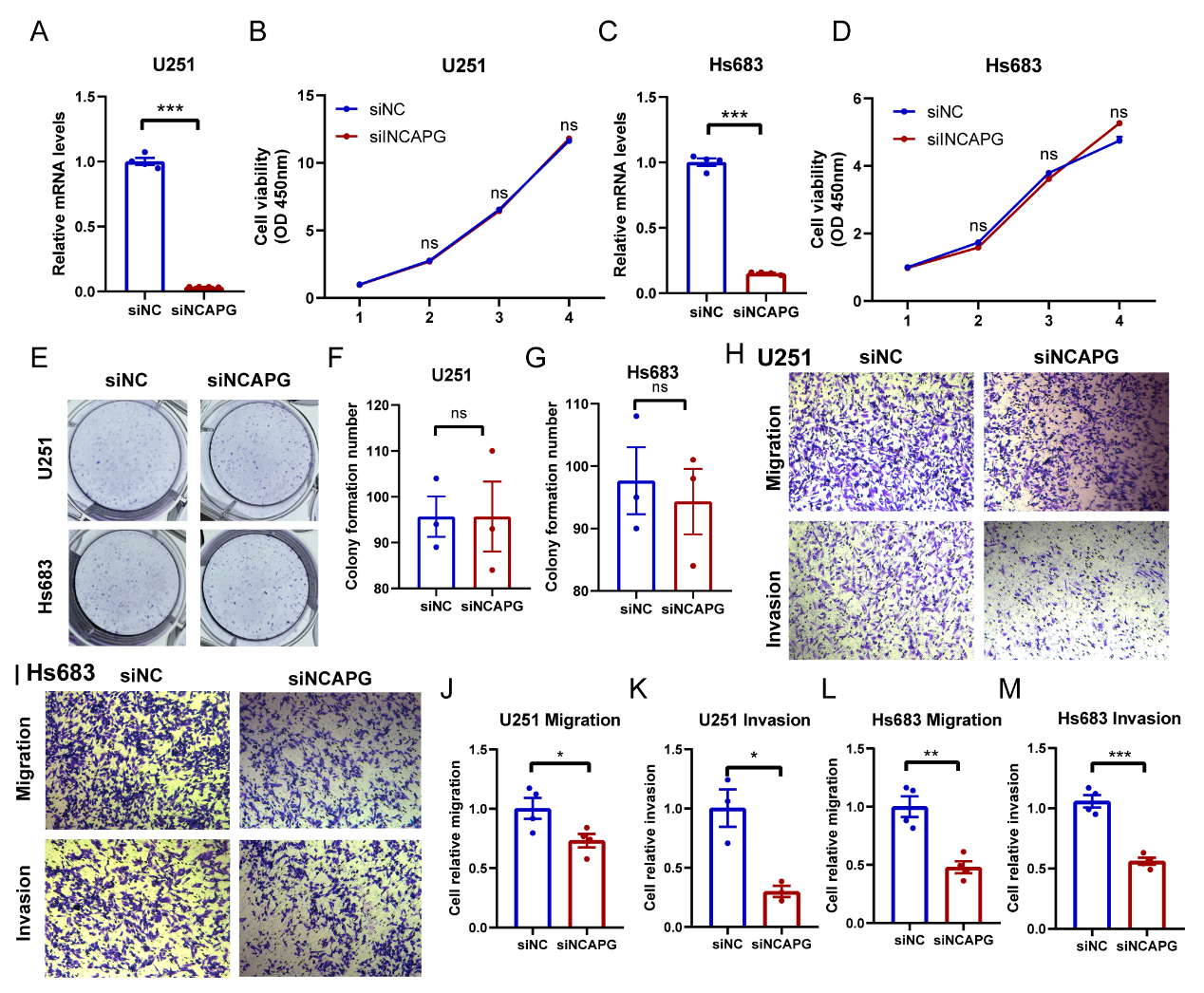


Figure S11. Effects of NCAPG knockdown on the proliferation, migration, and invasion of glioma cells. (A, C) qPCR analysis of NCAPG mRNA expression in U251 (A) and Hs683 (C) cells following siRNA transfection. (B, D) Cell viability assessed by CCK-8 assay in U251 (B) and Hs683 (D) cells (mean ± SEM, n = 6). (E-G) Colony formation assays performed in U251 and Hs683 cells. (H, I) Representative images of Transwell migration and invasion assays in U251 (H) and Hs683 (I) cells. (J-M) Quantification of cell migration and invasion numbers in the siNC and siNCAPG groups (mean ± SEM, n = 3). ns, not significant; *, P < 0.05. **, P < 0.01; ***, P < 0.001.
